# Rewiring of the 3D genome during acquisition of carboplatin resistance in a triple-negative breast cancer patient-derived xenograft

**DOI:** 10.1038/s41598-023-32568-7

**Published:** 2023-04-03

**Authors:** Mikhail G. Dozmorov, Maggie A. Marshall, Narmeen S. Rashid, Jacqueline M. Grible, Aaron Valentine, Amy L. Olex, Kavita Murthy, Abhijit Chakraborty, Joaquin Reyna, Daniela Salgado Figueroa, Laura Hinojosa-Gonzalez, Erika Da-Inn Lee, Brittany A. Baur, Sushmita Roy, Ferhat Ay, J. Chuck Harrell

**Affiliations:** 1 Department of Biostatistics, Virginia Commonwealth University, Richmond, VA 23298 USA; 2 Department of Pathology, Virginia Commonwealth University, Richmond, VA 23284 USA; 3 Department of Biology, University of Richmond, Richmond, VA 23173 USA; 4 Department of Biochemistry, Virginia Commonwealth University, Richmond, VA 23284 USA; 5 C. Kenneth and Dianne Wright Center for Clinical and Translational Research, Virginia Commonwealth University, Richmond, VA 23298 USA; 6 Center for Cancer Immunotherapy and Autoimmunity, La Jolla Institute for Immunology, La Jolla, CA 92037 USA; 7 Department of Pediatrics, UC San Diego—School of Medicine, La Jolla, CA 92093 USA; 8 Wisconsin Institute for Discovery, University of Wisconsin-Madison, Madison, WI 53715 USA; 9 Department of Biostatistics and Medical Informatics, University of Wisconsin-Madison, Madison, WI 53792 USA

**Keywords:** Breast cancer, Cancer genomics, Computational biology and bioinformatics

## Abstract

Changes in the three-dimensional (3D) structure of the genome are an emerging hallmark of cancer. Cancer-associated copy number variants and single nucleotide polymorphisms promote rewiring of chromatin loops, disruption of topologically associating domains (TADs), active/inactive chromatin state switching, leading to oncogene expression and silencing of tumor suppressors. However, little is known about 3D changes during cancer progression to a chemotherapy-resistant state. We integrated chromatin conformation capture (Hi-C), RNA-seq, and whole-genome sequencing obtained from triple-negative breast cancer patient-derived xenograft primary tumors (UCD52) and carboplatin-resistant samples and found increased short-range (< 2 Mb) interactions, chromatin looping, formation of TAD, chromatin state switching into a more active state, and amplification of ATP-binding cassette transporters. Transcriptome changes suggested the role of long-noncoding RNAs in carboplatin resistance. Rewiring of the 3D genome was associated with TP53, TP63, BATF, FOS-JUN family of transcription factors and led to activation of aggressiveness-, metastasis- and other cancer-related pathways. Integrative analysis highlighted increased ribosome biogenesis and oxidative phosphorylation, suggesting the role of mitochondrial energy metabolism. Our results suggest that 3D genome remodeling may be a key mechanism underlying carboplatin resistance.

## Introduction

High-throughput chromosome conformation capture (Hi-C) technology provides information on multiple levels of 3D chromatin organization^1^. These include chromatin loops, Topologically Associating Domains (TADs), and A/B (active/inactive) compartments, reviewed in^2,3^. At the kilobase scale, chromatin loops (local maxima pixels on Hi-C chromatin interaction maps) connect gene promoters with distal enhancers, promoting regulation of gene expression^4–6^. At the megabase scale, TADs represent regions on the linear genome that are highly self-interacting^7,8^.

Disruption of chromatin interactions due to copy number variants and even single nucleotide polymorphisms has been reported to promote enhancer hijacking^9,10^, fusion of TADs^11,12^, creation or destruction of sub-TADs within existing TAD boundaries^13,14^, and/or switching chromatin states between active and inactive conformations^1,7^. These events are frequent in cancer, leading to coordinated expression of oncogenes and/or silencing of tumor suppressors^15,16^.

Breast cancer is now the second leading cause of cancer-related deaths in the US, and the incidences of breast cancer continue to rise^17^. About 15–20% of breast cancers present with an aggressive hormone receptor-negative (triple negative breast cancer, TNBC) phenotype^18^. TNBC cancers are characterized by rapid proliferation rates and high metastatic propensity, and they are associated with the worst outcome of all breast cancer subtypes. Unlike estrogen receptor, progesterone receptor, or human epidermal growth factor receptor 2 positive breast cancers, TNBCs cannot be treated with endocrine therapies or HER2-targeted agents (Trastuzumab, Pertuzumab), and chemotherapies are standard of care. The addition of carboplatin (CBDCA, cis-Diammine-1,1-cyclobutane dicarboxylate platinum) to neoadjuvant therapy increases the proportion of patients achieving a pathological complete response^19^. Unfortunately, drug resistance can occur, and 90% of treatment failures in metastatic cancers are attributed to chemoresistance (i.e., drug resistance). While molecular mechanisms of drug resistance have been explored^20^, the association between 3D changes and drug resistance remain underexplored.

**Figure 1:**
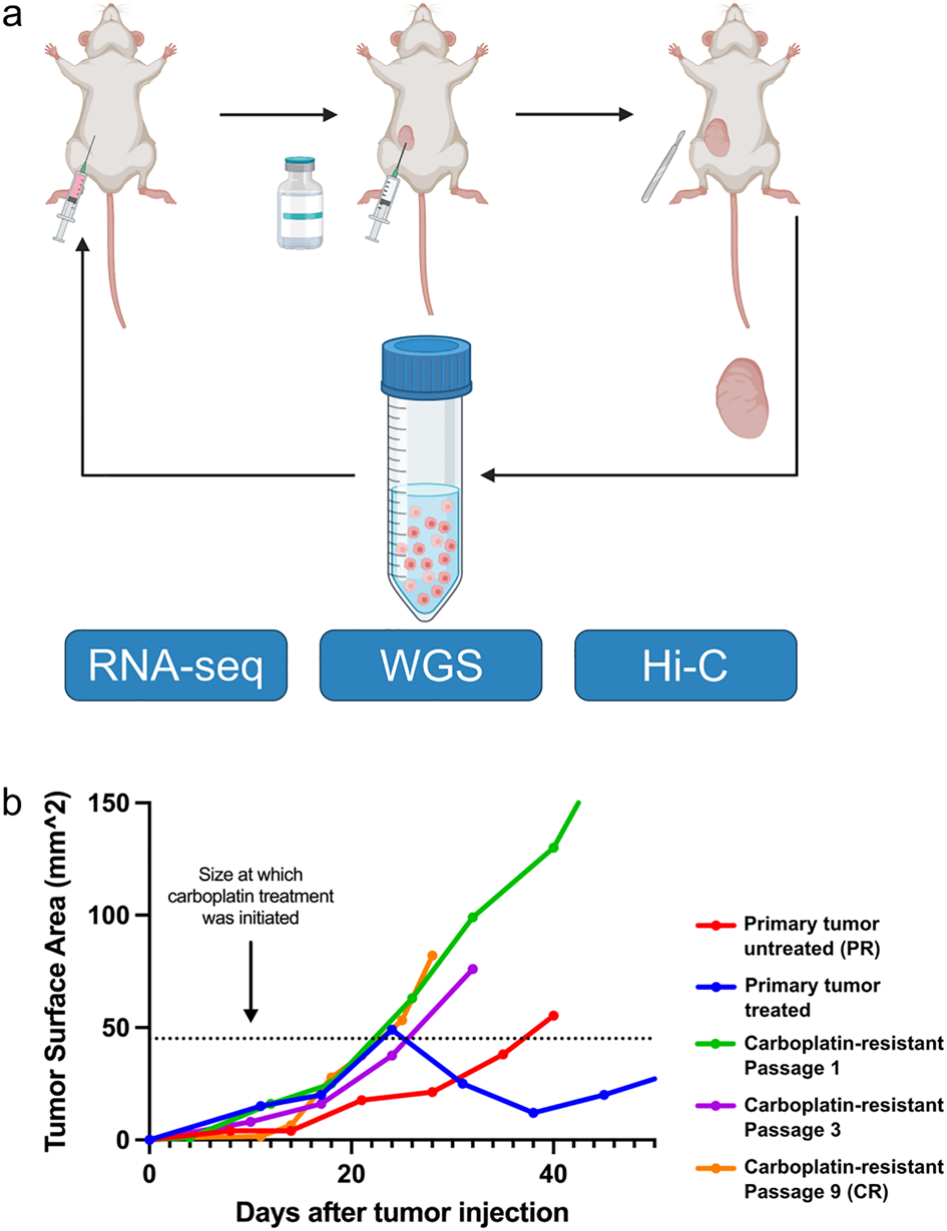
Development of a PDX-model of acquired carboplatin resistance in triple-negative breast cancer. (**a**) UCD52 PDX tumors were grown in female NSG mice. Once tumors reached 25–50 mm^2^ in size, mice were treated with carboplatin. After tumor growth resumed, tumors were harvested, prepared into a single-cell suspension, and passaged into a new recipient mouse. Single-cell suspensions were also used for Hi-C, WGS, and RNA-seq. (**b**) Plot depicts growth rates of untreated and treated primary (PR) UCD52 tumors as well as passage number 1, 3, and 9 carboplatin resistant (CR) tumors. N = 1 or 2 per passage number. Image created with BioRender.com.

In this study, we characterized the 3D genome organization changes during TNBC cancer progression from primary (PR) drug-sensitive state to a carboplatin-resistant (CR) state. We utilized the TNBC patient-derived xenograft (PDX) model UCD52, which, among other PDXs, is known to faithfully recapitulate the heterogeneity and genomics/transcriptomic profiles of human disease^21^. The carboplatin-resistant state was established through the progressive passaging of PDX tumors under treatment until tumors continued growing in the presence of the drug (Fig. 1). We performed replicated Hi-C experiments, RNA-seq, and whole-genome sequencing (WGS) to understand the interplay between 3D structure, gene expression, and copy number variant changes associated with carboplatin resistance. The carboplatin-resistant state was characterized by the genome-wide increase in short-range chromatin interactions (< 2 Mb), chromatin loops, and more topologically associating domains (TADs). Gene expression changes highlighted near-complete shutdown of drug metabolism pathways and activation of many aggressiveness-, metastasis-, and other cancer-related signatures. A number of long noncoding RNAs associated with cancer-related phenotypes and functions were significantly upregulated. Analysis of copy number variation confirmed these findings and highlighted the amplification of ATP-binding cassette (ABC) transporters known for multidrug resistance. Integrating different omics layers strengthened the evidence for amplification of ABC transporters and activation of cancer- and drug resistance-related genes/pathways, and the role of mitochondrial energy metabolism in carboplatin resistance. Our study provides a unique multiomics dataset and characterizes the 3D genomic reorganization in carboplatin resistance in TNBC.

## Results

### Carboplatin resistance-associated gene expression changes may promote more aggressive phenotype

**Figure 2:**
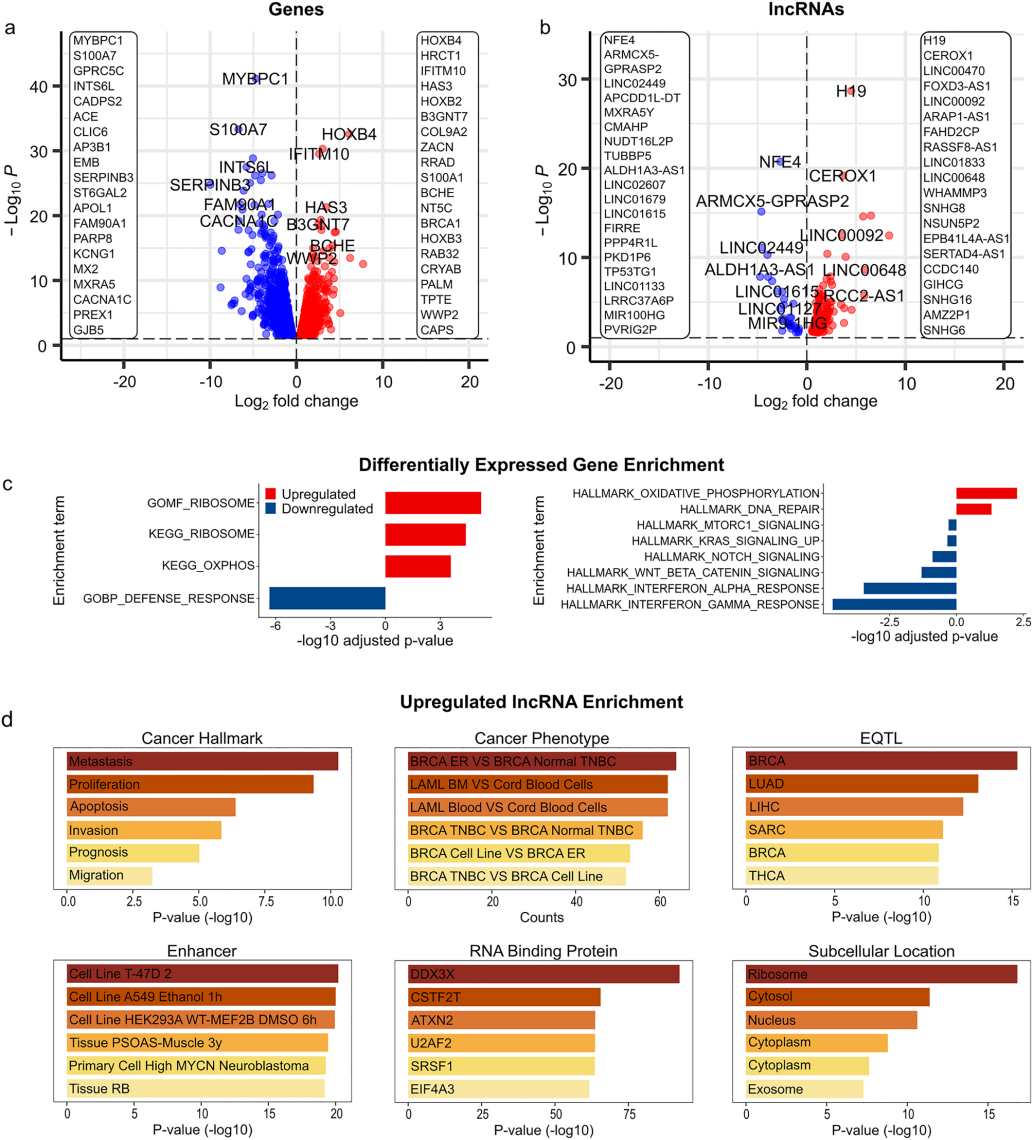
Differentially expressed transcripts and their functional significance. Differentially expressed protein-coding genes (**a**) and lncRNAs (**b**). Red/blue colors correspond to genes up- or downregulated in the CR condition. Top 20 most significant up- and downregulated transcripts are listed in the corresponding panels and selected transcripts are highlighted. (**c**) Most significant GSEA enrichments using KEGG, Gene Ontology, and Hallmark MSigDb collections. (**d**) Functions, phenotypes, and signatures enriched in lncRNAs upregulated in carboplatin resistance. Each panel corresponds to enrichment categories from the LncSEA analysis. All panels show −log10(p-value) except “Cancer Phenotype” showing gene counts.

Dysregulated gene expression in cancer is tightly coupled to alterations in 3D structure^15,16^. To better understand transcriptome changes in carboplatin resistance, we performed RNA-seq comparing gene expression changes between primary tumor (PR) and carboplatin-resistant (CR) state. We observed minimal mouse read contamination, 3.83% and 1.93% for UCD52PR/UCD52CR, respectively. We detected an approximately equal number of up-and downregulated transcripts (2052 up and 1933 down, edgeR FDR = 0.1) in the CR condition (Supplementary Table S1). Considering protein-coding genes, *HOXB4* (homeobox B4) was the most significantly upregulated (log2FC = 6.14, FDR = 7.72E−30), and its expression was associated with multidrug resistance of human myelogenous leukemia^22^ (Fig. 2a). Among the top 20 upregulated genes, we observed *BRCA1* (log2FC = 3.85, FDR = 2.70E−13), mutations of which were associated with a more aggressive TNBC phenotype^23^. Other upregulated genes were similarly associated with cancer aggressiveness, metastasis, and other oncogenic properties that may facilitate resistance in the CR condition. We observed more downregulated protein-coding genes (1837, vs. 1637 upregulated), with *MYBPC1* (myosin binding protein C1) being the most significantly downregulated (log2FC = − 4.71, FDR = 5.58E−38). Together with 43 other downregulated genes, it was among the list of 257 downregulated genes in purified TNBC cells identified by Komatsu et al.^24^ (hypergeometric *p* value < 1.00E−3). These results suggest that carboplatin resistance-associated gene expression changes may promote aggressive cancer phenotype.

To understand the functional significance of the CR transcriptome changes, we performed GSEA analysis using KEGG pathways and MSigDB collections^25^ (Supplementary Table S1). Analysis of the “C2 curated gene sets” collection identified several cancer-related processes, such as the enrichment of CR-downregulated genes in metastasis-downregulated “Jaeger Metastasis Down” signature (93 genes, NES = − 2.34, FDR = 1.79E−4) (Supplementary Table S1). We also observed enrichment of CR-downregulated genes in adhesion-related signatures, such as “Onder CDH1 Targets Down” (160 genes, NES = − 2.25, FDR = 4.27E−5). These genes were downregulated in HMLE cells (immortalized non-transformed mammary epithelium) after E-cadhedrin (*CDH1*) knockdown by RNAi. Loss of E-cadherin promotes metastasis by disrupting intercellular contacts^26^; consequently, our observations suggest increased metastatic potential in the CR condition. We also observed several downregulated cancer-related pathways and hallmark signatures, such as “Sana TNF Signaling Up” (33 genes, NES = − 2.65, FDR = 3.19E−5); however, the “DNA Repair” signature was upregulated (NES = 1.80, FDR = 4.91E−2). These results further strengthen our observation that aggressiveness and metastatic signatures may be upregulated in carboplatin resistance.

### Ribosomal and oxidative phosphorylation/metabolic genes are upregulated in carboplatin resistance

Among KEGG pathways, “Ribosome” was the most significant (37 genes, Normalized Enrichment Score (NES) = 2.66, FDR = 4.01E−5), (Fig. 2c). Similarly, “Structural Constituent of Ribosome” was among the most significant ontologies in the “C5: ontology gene sets” MSigDb’s collection (62 genes, NES = 2.79, FDR = 5.84E−6). “Oxidative phosphorylation” was another KEGG pathway upregulated in CR and enriched in NADH-Ubiquinone Oxidoreductase Subunit encoding genes, ATP Synthase genes, and others (24 genes, NES = 2.64, FDR = 2.67E−4). “Oxidative Phosphorylation” gene set was similarly upregulated in the “H: hallmark gene sets” MSigDb’s collection (50 genes, NES = 2.04, FDR = 4.59E−3). Importantly, this pathway has recently been described as a mediator of resistance to chemotherapy^27^. These results suggest that protein synthesis and metabolic activities may be enhanced in carboplatin resistance.

### Immune signature downregulation in carboplatin resistance

“Defense Response” was the most significant gene ontology enriched in downregulated genes (332 genes, NES = − 2.33, FDR = 4.10E−7), (Fig. 2c, Supplementary Table S1). Similarly, “Immune Response” ontology was enriched in 328 downregulated genes (NES = − 2.19, FDR = 4.10E−7). Analysis of the MSigDB’s “C2 curated gene sets” collection identified “Sana Response to IFNG Up” signature among the most significant enriched in 45 downregulated genes (NES = − 2.60, FDR = 1.96E−6). The “H hallmark gene sets” analysis also identified “Interferon Gamma Response” enriched in 77 downregulated genes (NES = − 2.39, FDR = 1.97E−5) and “Interferon Alpha Response” signatures (48 genes, NES = − 2.30, FDR = 2.88E−4). Overexpression of IFN-inducible genes was observed in chemo-responder PDX models of TNBC with no changes in non-responders^28^; consequently, downregulation of interferon signatures in the CR condition may indicate a favorable condition for carboplatin resistance development.

### Long noncoding RNAs are overrepresented in carboplatin resistance

Considering transcript types, we observed significantly more lncRNA being upregulated in CR (317 vs. 54 downregulated, Chi-square *p* value = 1.24E−42) (Supplementary Table S1). *H19* (imprinted maternally expressed transcript), the first discovered lncRNA^29^ was the top significantly upregulated (Fig. 2b) and is known to promote cancer stemness and paclitaxel resistance^30^. Literature review identified other top up- and downregulated lncRNAs also promoting aggressiveness and drug resistance in breast and other cancers. Paralleling RNA-seq analysis observations, *CEROX1* (cytoplasmic endogenous regulator of oxidative phosphorylation 1), the second most upregulated lncRNA, suggested the role of oxidative phosphorylation. To understand the collective effect of lncRNAs, we used LncSEA^31^ (Supplementary Table S1). LncSEA analysis identified enrichment of upregulated lncRNAs in “Metastasis” (7 lncRNAs, hypergeometric FDR = 3.63E−10), “Proliferation” (10 lncRNAs, FDR = 1.58E−9) and similar cancer hallmark signatures (Fig. 2d), suggesting increased aggressiveness of cancer cells in carboplatin resistance. “Breast cancer ER+ vs. Breast cancer Normal TNBC” (64 lncRNAs, FDR = 1.64E−16) and other breast cancer-related cancer phenotype signatures shared the largest number of upregulated lncRNAs. Breast cancer specificity was also reflected in the enrichment of upregulated lncRNAs in BRCA-associated cis-eQTLs (67 lncRNAs, FDR = 1.61E−14) and in enhancers in T-47D breast cancer cell line (47 lncRNAs, FDR = 2.07E−18). Similar to the analysis of protein-coding genes, “Ribosome” was the most significant subcellular location enriched (15 lncRNAs, FDR = 1.50E−16), although “Cytoplasm” had the largest number of lncRNAs (53 lncRNAs, FDR = 6.48E−9). The small number of downregulated lncRNA transcripts (54 total, 30 had annotations) was insufficient for all enrichment analysis; yet, 21 downregulated lncRNAs were enriched in targets of *TP63* (FDR = 1.00E−5). These results suggest that carboplatin-resistant lncRNAs may support protein-coding transcripts in enhancing metabolic activities and promoting aggressive cancer phenotype.

### WGS data reveals twice as many deletions than amplifications in carboplatin resistance

**Figure 3:**
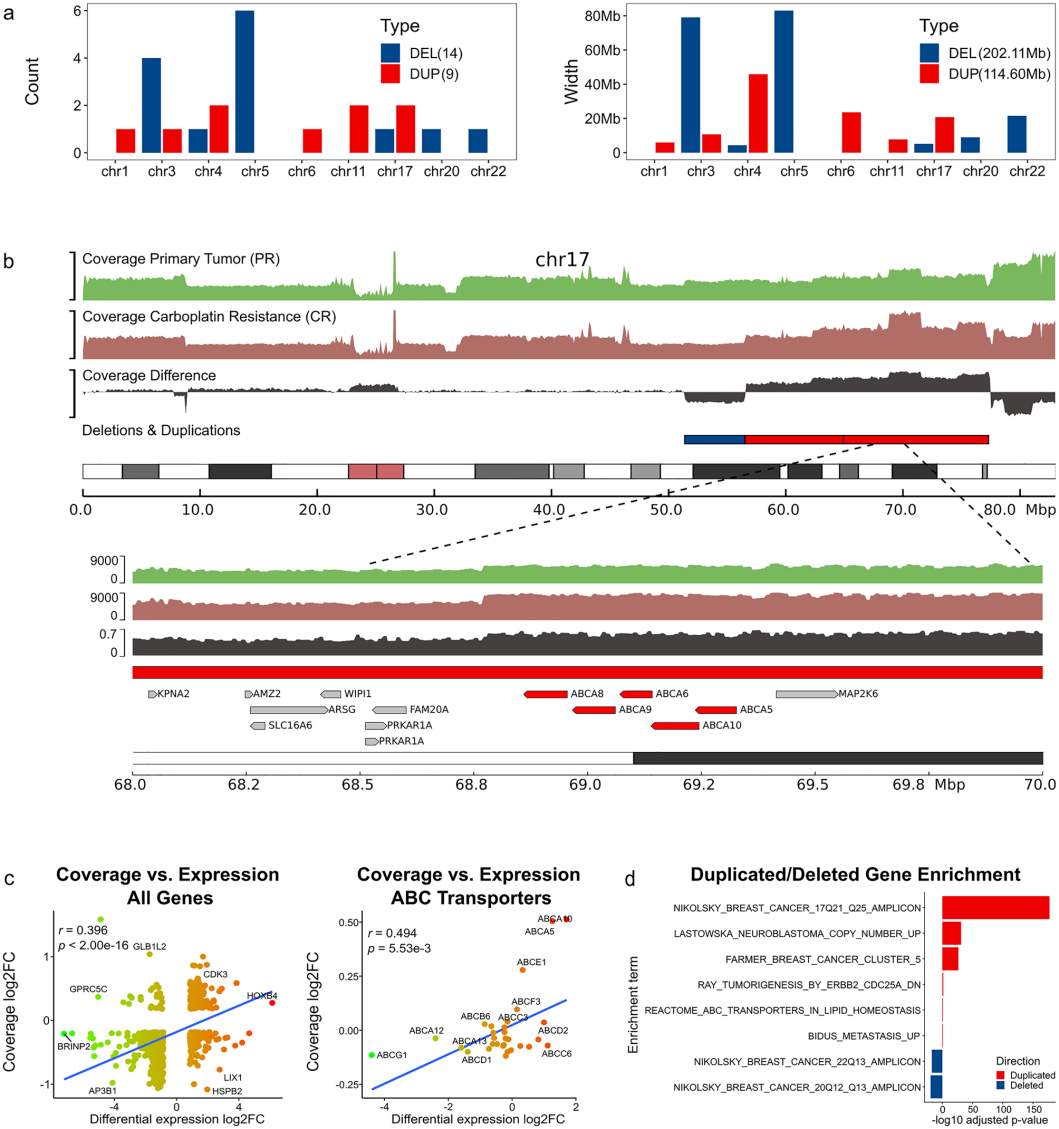
Whole Genome Sequencing coverage differences between the CR and PR conditions. (**a**) Count and size of large deletions and duplications in the CR condition identified by the Circular Binary Segmentation algorithm. (**b**) An example of large deletions and duplications on chromosome 17 with a portion containing ABC transporters zoomed-in. (**c**) Correlation of gene expression and coverage changes, genomewide and ABC transporters only. Genes with small changes (less than 1SD of the change distribution) were excluded. (**d**) Gene sets from MSigDb enriched in deleted (blue) or duplicated (red) genes.

Cancer-specific copy-number alterations have been associated with changes in chromatin structure^13,32^. To understand the effect of large-scale copy-number alterations (deletions and duplications), we compared WGS data between the CR and PR conditions. We found 10.34% and 15.49% mouse reads contamination in the UCD52PR/UCD52CR data, respectively. The coverage between WGS and Hi-C data was positively correlated (Mean Spearman correlation of 10 kb-binned coverage for PR/CR = 0.699/0.671, respectively). As expected for a highly rearranged carboplatin resistant genome, the CR genome had more reads with low mapping quality and pairs mapping to different chromosomes (Supplementary Table S2, Supplementary Figure S1). Chromosomes 3, 5, 20, and 22 had fewer than average coverage indicative of large deletions in CR. In contrast, chromosomes 4, 6, and 17 showed higher than average coverage in CR, suggestive of large duplications. The comparison of mitochondrial chromosome coverage similarly identified nearly double coverage (1.79X) in the CR as compared with the PR condition. Indeed, circular binary segmentation of coverage log2 ratio (coverage differences between the CR and PR conditions) identified these chromosomes as having large genomic variants (Fig. 3a,b, Supplementary Figure S2). Similarly, consensus SV calls using delly, lumpy, and breakdancer showed more CR-specific deletions and fewer duplications as compared with the PR condition (Supplementary Table S2, Supplementary Figure S3). In total, 202.11 Mb genomic DNA was deleted, and 114.60 Mb was duplicated in the CR condition when compared to PR, corresponding to 6.52% and 3.70% of the total genome size, respectively. Similarly, out of all genes overlapping copy number variant regions 71.64% of them were located in copy number-low regions, while 28.36% were in copy number-high regions. These results suggest that the carboplatin-resistant genome undergoes significant rearrangements with enrichment towards deletions.

We then interrogated the influence of copy number variation on gene expression. Correlating gene expression changes and their coverage changes of corresponding loci, we observed a significant positive correlation (Pearson correlation = 0.396, *p* value < 2.00E−16, see Methods) (Fig. 3c). Focusing on ABC transporters (described in the following section), we similarly observed a high correlation (Pearson correlation = 0.494, *p* value = 5.53E−3). These results highlight general concordance between genomic copy number variation and transcriptome changes in carboplatin resistance.

### ABC transporters are amplified in carboplatin resistance

Genes deleted in CR were enriched in “Nikolsky Breast Cancer 20q12-q13” (43 genes, hypergeometric FDR = 3.64E−20) and “Nikolsky Breast Cancer 22q13” (16 genes, FDR = 5.41E−18) regions (Fig. 3d, Supplementary Table S2). Conversely, genes on chromosome 17 region “Nikolsky Breast Cancer 17q21-q25” were amplified in CR and contained ATP-binding cassette (ABC) transporters (*ABCA5/ABCA6/ABCA8/ABCA9/ABCA10*) among them (Fig. 3b, 172 genes total, FDR = 7.39E−178). Similarly, genes in “Farmer Breast Cancer Cluster 5”, which is a subset of the 17q21-25 genes containing ABC transporters^33^, were amplified (19 genes, FDR = 3.07E−27). We performed GSEA analysis on genes ranked by coverage difference (Methods) and found similar enrichments (Supplementary Table S2). ABC transporters are known to promote chemoresistance via drug efflux and contribute to cancer development and metastasis via other mechanisms^34–36^. Paralleling our observation of upregulated lncRNAs, the role of noncoding RNAs in regulating ABC transporters started to emerge^37^. These results suggest that amplification of ABC transporters may be a hallmark of genome rearrangement in carboplatin resistance.

### Chromatin conformation changes show stronger short-range interactions in carboplatin resistance

**Figure 4:**
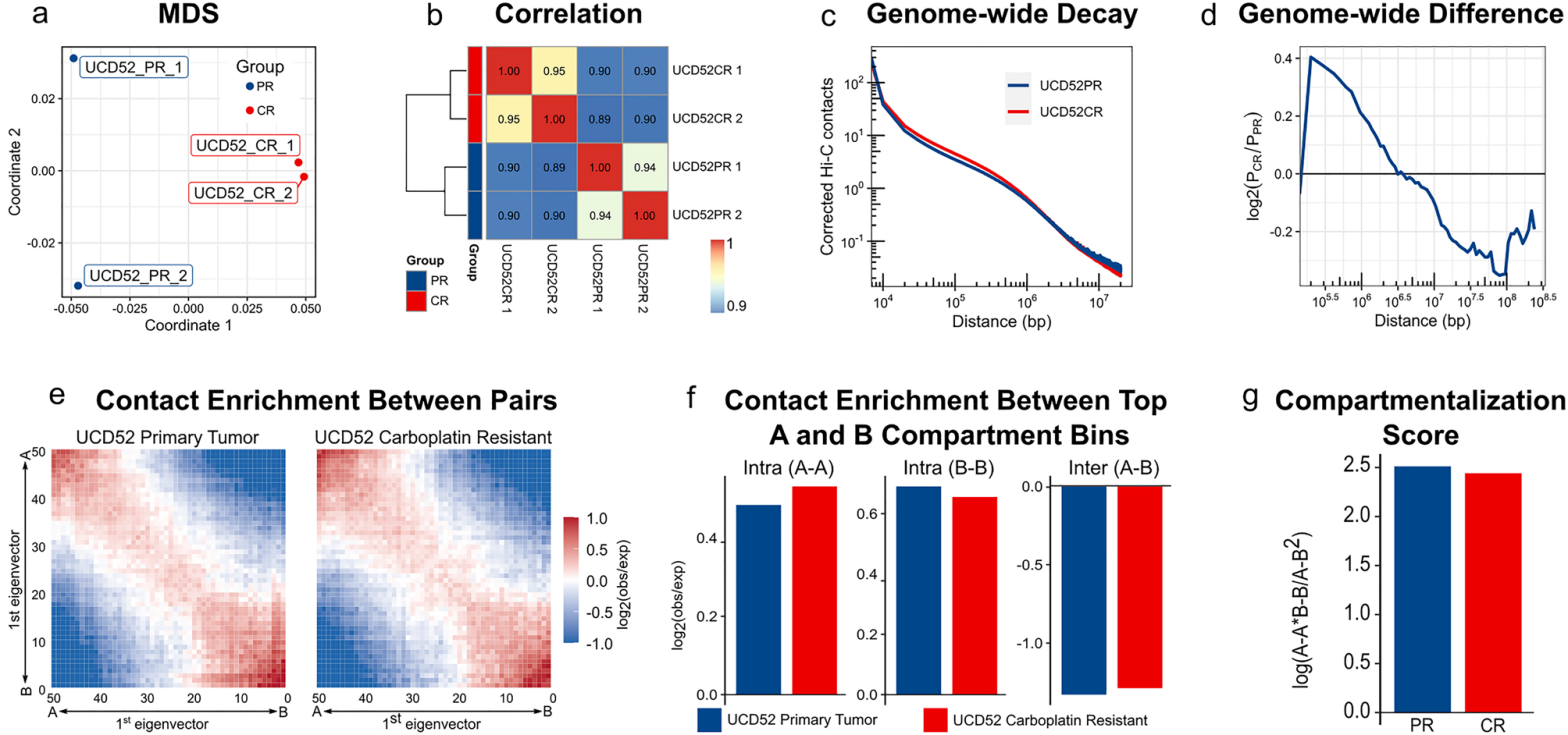
Replicability and compartmentalization changes in carboplatin resistance. (**a**) Multi-dimensional scaling (chromosome-specific HiCRep measures, averaged for each pairwise comparison) and (**b**) a heatmap of Pearson Correlation Coefficients for replicates at 1 Mb resolution. (**c**) Distance-dependent chromatin interaction decay curves (X-axis—distance on log10 scale, Y-axis—corrected chromatin contacts on log10 scale) and (**d**) differences between them (Y-axis—log2 ratio of contact probabilities for CR vs. PR conditions). (**e**) Compartmentalization saddle plots (500 kb resolution), (**f**) contact enrichments between top A and B compartment bins (Methods), and (**g**) overall compartmentalization score in the CR versus PR condition comparison.

To understand the role of 3D genomic changes in carboplatin resistance, we performed Hi-C sequencing with two replicates per condition yielding $\sim 1.4$ billion read pairs; their quality metrics and the proportion of mouse reads were comparable (Supplementary Table S3). Reproducibility assessment of replicates using HiCRep demonstrated high concordance within replicates (Fig. 4a). Similarly, correlation of Hi-C matrices at 1 Mb resolution showed high concordance within conditions (Pearson Correlation Coefficient (PCC) $\sim 0.95$) and lower concordance between conditions (PCC $\sim 0.90$, Fig. 4b). Condition-specific replicates were merged for downstream processing and analyzed at 10 kb resolution. The differential chromatin contact maps are shown in Supplementary Figure S4. Comparison of the decay curves identified a larger number of short-range interactions within the 2 Mb distance range, the physiological size of Topologically Associating Domains (TADs) (Fig. 4c,d). These observations suggest that genome-wide short-range interactions and TADs may be strengthened in CR.

### More chromatin compartments switch to an active state in carboplatin resistance

**Figure 5:**
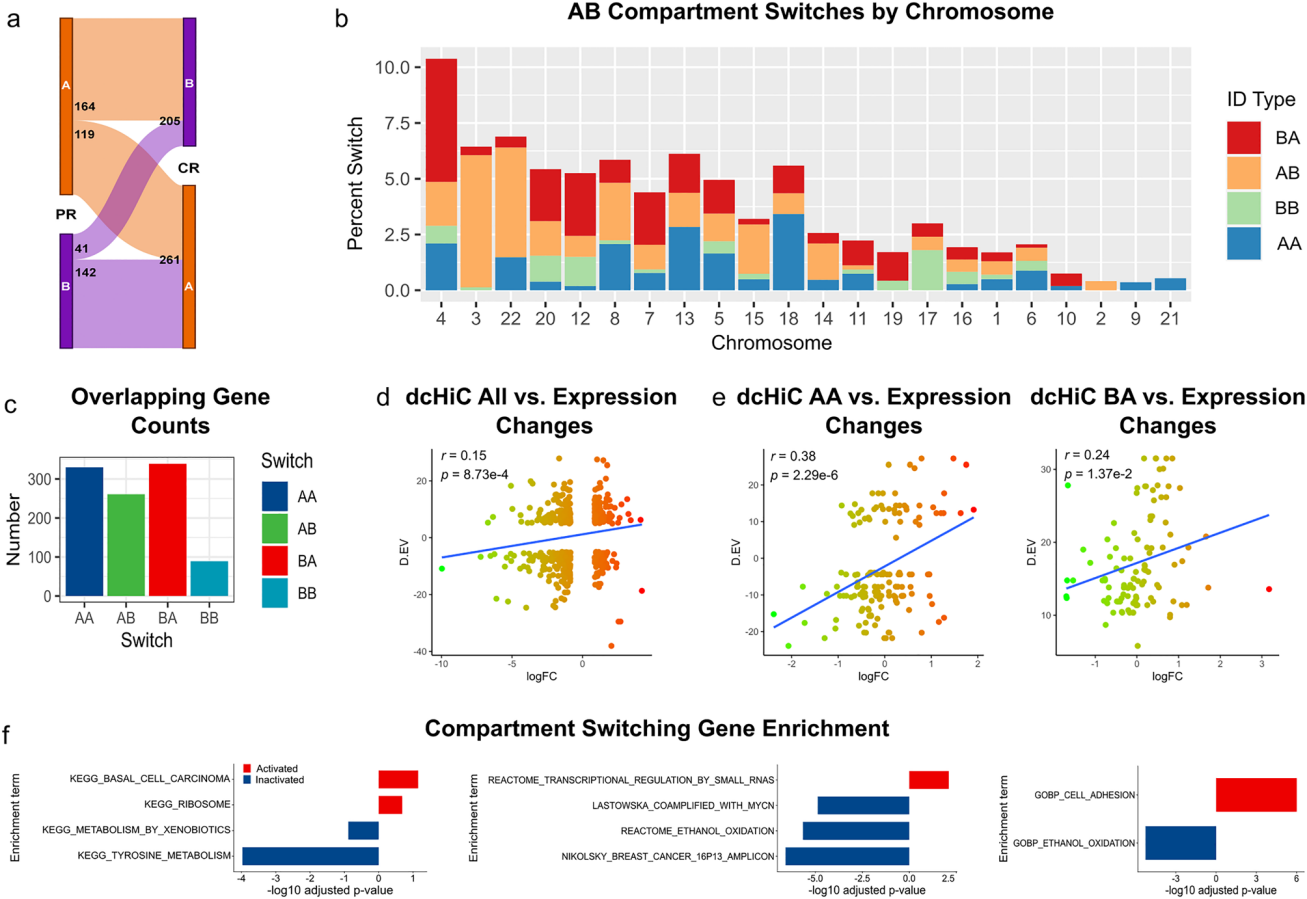
Chromatin state changes in carboplatin resistance. (**a**) Genomewide flow chart and (**b**) chromosome-specific proportions of the genome switching states between active A and inactive B compartments in the CR versus PR comparison at FDR < 0.3. (**c**) Number of genes overlapping chromatin state switching regions. (**d**) Correlation between gene expression- (X-axis) and chromatin state (eigenvector) changes (Y-axis). Only changes larger than 1SD or (**e**) changes within AA or BA switches were considered. (**f**) Most significant GSEA enrichments using KEGG, curated gene sets, and Gene Ontology MSigDb collections.

3D structural changes in cancer genomes have been described at all levels of 3D genome organization^16^. We first investigated differences in open (transcriptionally active) and closed (inactive) genomic compartments (A and B, respectively). We quantified genome-wide differences in A-A and B-B interactions as described^38^ and found increased A-A interactions with the parallel decrease in B-B interactions in the CR condition (Fig. 4e,f). Inter-compartment interactions (A-B, B-A) and the global compartmentalization score were largely unchanged (Fig. 4g). To get a finer insight into the A/B compartment changes, we used dcHi-C which utilizes quantile normalized compartment scores calculated from eigenvector decomposition and a multivariate distance measure to identify statistically significant changes in compartmentalization among multiple contact maps^39^. Furthermore, to avoid considering eigenvectors capturing chromosomal arms, dcHiC checks for eigenvector correlation with gene density and GC content and anticorrelation with chromosome length (Supplementary Table S3). We compared genome-wide dcHiC-selected eigenvectors between the CR and PR conditions and found the majority of the genome preserving its compartment state when transitioning into the carboplatin-resistant state (AA/BB states occupying 44.61%/45.29% of the genome, respectively, Supplementary Figure S5A,B,C). These compartments were not associated with structural variants likely due to their large genomic footprint making permutation analysis infeasible. Approximately 10% of the genome switched compartments, with 5.38% and 4.72% of the genome undergoing AB/BA compartment switch, respectively. We then compared statistically significant compartment changes (FDR < 0.3). Besides AB and BA compartment switches, we note that compartmentalization magnitude differences may be significant within regions of the same compartment state (AA and BB) (Fig. 5a). Of all significant changes, 36.60% and 31.47% were AB/BA switches, and 22.84%/9.09% significant changes were AA/BB magnitude differences, respectively (Supplementary Figure S5D). Chromosome 4 had the largest proportion of significant BA compartment switches (5.52%) (Fig. 5b). On the contrary, chromosomes 3 and 22 had the largest number of significant AB switches (5.93% and 4.92%, respectively). Together with strengthening o short-range interactions, more changes in active compartments (54.31% AA and BA changes) than inactive compartments (45.69% AB and BB changes) suggest increased transcriptional activity in carboplatin resistance.

### Compartment changes lead to the activation of many genes

We focused on genes overlapping significant compartment switches. Expectedly, the total number of genes overlapping significant BB regions was low (89 genes), reflecting the transcriptionally inactive state of B compartments. In contrast, the number of genes in regions switching from B (inactive) in PR to A (active) in the CR compartment state was the highest (339 genes), followed by genes in the AA and AB switches (330 and 261, respectively, Fig. 5c). In summary, we observed 669 genes in chromatin regions switching to the active state (AA and BA compartment switches) as compared with 350 genes switching into the inactive state (BB and AB switches). These results suggest that compartment changes in carboplatin resistance led to the activation of many genes.

We hypothesized that changes in AB compartments may correspond to changes in gene expression. We observed positive correlation between eigenvector changes and gene expression changes (PCC = 0.15, *p* value = 8.73E−4, Fig. 5d). This correlation was highest for genes in AA compartments (PCC = 0.38, *p* value = 2.29E−6) and BA compartments (PCC = 0.24, *p* value = 1.37E−2) (Fig. 5e), suggesting that changes in active chromatin compartments are associated with gene expression changes. Conversely, correlation within AB compartments was low (PCC = 0.12, *p* value = 3.04E−1) and even negative in BB compartment changes (PCC = − 0.26, *p* value = 2.42E−1) (Supplementary Figure S5E). These observations support the notion that carboplatin resistance-associated interaction strength changes occur in transcriptionally active chromatin regions; consequently, we expect genes associated with compartment changes to be enriched in pathways and gene signatures identified in RNA-seq analysis.

### Metabolism genes switch into the inactive state in carboplatin resistance

To understand the collective effect of compartment changes, we ranked genes by the maximum eigenvector difference. GSEA analysis of KEGG pathways identified the decreased activity (enrichment in downregulated genes) of amino-acid-, sugar-, and xenobiotic metabolic pathways, such as “Tyrosine metabolism” (8 genes, NES = − 2.27, FDR = 1.07E−4) (Fig. 5f, Supplementary Table S3). Activity of the “Basal cell carcinoma” pathway was increased (enriched in upregulated genes) in CR (17 genes, NES = 1.61, FDR = 7.07E−2), potentially reflecting more aggressive state of carboplatin-resistant cells. Analysis of the “C2” MSigDb category identified several “Nikolsky Breast Cancer Amplicon” signatures^40^ enriched in genes switching into inactive state. These genes were frequently colocalized with CR-specific deletions, confirming the notion that genomic rearrangements, gene expression, and compartment switches are related. We also observed downregulation of several metabolic pathways, such as “Ethanol Oxidation” (7 genes, NES = − 2.23, FDR = 2.21E−6). Notably, the “Transcriptional Regulation by Small RNAs” signature was switching into active state (67 genes, NES = 1.78, FDR = 7.79E−3), that, together with upregulation of lncRNAs from the RNA-seq analysis, suggest the role of noncoding transcripts in carboplatin resistance. Analysis of the “C5” MSigDb category (ontologies) identified activation of “Homophilic Cell Adhesion Via Plasma Membrane Adhesion Molecules” genes (76 genes, NES = 2.22, FDR = 9.82E−7) ontology and inactivation of “Ethanol Oxidation” (7 genes, NES = − 2.25, FDR = 5.39E−6) and other metabolic functions. These results suggest decreased activity of metabolic pathways in carboplatin resistance.

### Longer-range and CTCF-independent loops are enriched in the CR condition

**Figure 6:**
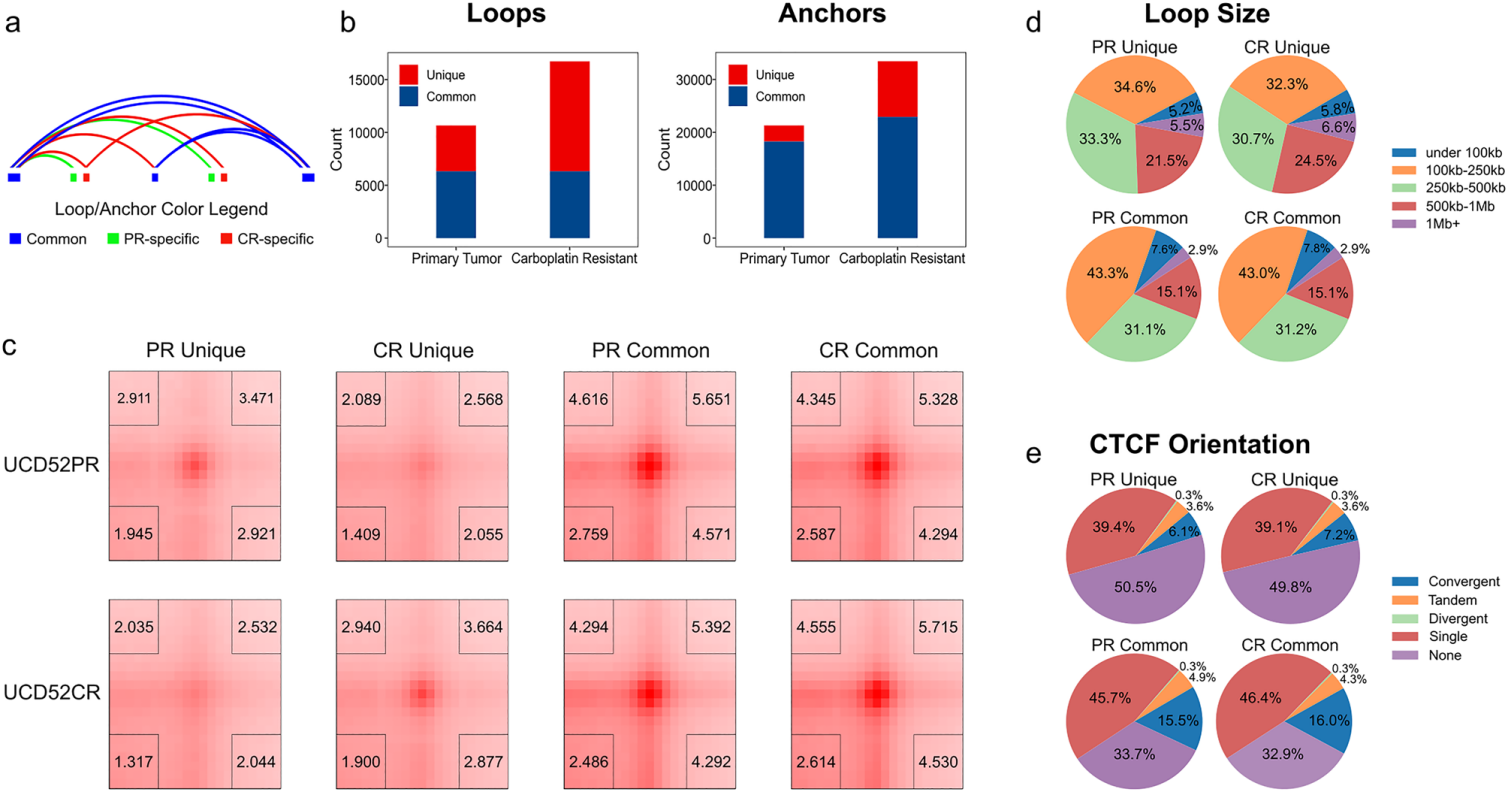
Condition-specific and common loops and anchors. (**a**) Differences between loops and anchors. Common loops (blue arcs) and PR/CR-specific loops (green/red arcs) may share anchors that are considered common (blue rectangles), while the other, loop-specific anchors, are considered PR/CR-specific (green/red rectangles). Adjacent anchors are considered in the same category. (**b**) Counts of the condition-specific and common loops and anchors. (**c**) Aggregate Peak Analysis of condition-specific and common loops (X-axis) in the condition-specific matrices (Y-axis). Corner numbers correspond to center-to-corner ratios. (**d**) Size range comparison of loop size distributions, (**e**) Proportions of loops with various CTCF configurations at boundaries.

Chromatin loops are considered the primary mechanism of enhancer-promoter interactions. We considered loops detected by Mustache^4^ in the PR and CR conditions. Loops are defined as pairs of interacting regions (i.e., loop anchors), while anchors are individual regions. We separated them into common and condition-specific loops and anchors, allowing ± 1 bin flank (neighbor regions are considered while computing overlap). Figure 6a illustrates common loops with adjacent anchors at one or both ends. It also demonstrates the difference between loops and anchors in that a condition-specific loop may have both common and condition-specific anchors (Supplementary Table S4).

Mustache detected $\sim 1.5$ times more loops in the CR condition as compared to the PR condition (16,716 vs. 10,652, results are reported at 10 kb resolution unless specified otherwise). Separating condition-specific and common loops identified more than 2 times unique loops in the CR condition as in the PR conditions (10,397 vs. 4333) (Fig. 6b). Comparing the proportions of common versus unique loops identified the majority of CR loops were unique (10,397 unique vs. 6319 common loops); the reverse was observed for PR loops (4333 unique vs. 6319 common loops). Similarly, anchor analysis identified more anchors in the CR condition, and the majority of them were CR-specific. These observations were consistent across resolutions. The PR-specific loop anchors were marginally enriched in the AB compartment switches (permutation *p* value = 2.80E−2), while the CR-specific anchors were enriched in both AB and BA compartment switches (*p* value < 1.00E−4), suggesting chromatin rewiring in compartment-switching regions in the CR condition. Integrating the PR- and CR-specific loop anchors with structural variations, we found them to be depleted in the condition-specific deletions (*p* value = 1.00E−2–1.00E−4) and deletions and duplications detected across both the PR and CR genomes (*p* value < 1.00E−4). These results suggest that structural variants may indirectly affect chromatin rewiring. The Aggregate Peak Analysis (APA) confirmed the enrichment of PR/CR-specific loops in the corresponding Hi-C matrices, while common loops showed enrichment in both conditions (Fig. 6c). With the observation of increased short-range interactions and chromatin switching into a more active state, these results suggest a major interactome rewiring via many unique loops in the CR condition.

We compared size of the PR/CR specific/common loops. Loops specific for either condition were significantly longer than the PR/CR-common loops (median size 310 kb in PR/330 kb in CR vs. 250 kb for common loops, 10 kb resolution, Wilcoxon *p* value < 1.04E−41, Supplementary Table S4). These observations were consistent across resolutions. The CR-specific loops were generally longer than the PR-specific loops at 10 kb resolution (median size 330 kb vs. 310 kb). Considering size ranges, we found a smaller proportion of condition-specific loops with a size under 100 kb and a larger proportion of loops larger than 1 Mb (Fig. 6d). These results suggest that most condition-specific loop interactions occur at longer distances.

CTCF is the most well-known protein enriched at loop anchors in convergent orientation. We first quantified the number of multiple, single, and adjacent CTCF motifs overlapping condition-specific and common anchors. Condition-specific anchors were less overlapping with CTCF motifs than common loops (Supplementary Table S4). Considering convergence types, we found that condition-specific loops had fewer loops with CTCF in convergent orientation and more loops without CTCF binding (Fig. 6e). While these results may indicate that condition-specific loops may be transitory, their aggregate peak analysis suggests they show a strong local enrichment indicative of strong loops specifically in the condition where they are reported. In summary, our observations suggest that condition-specific loops are less dependent on CTCF, longer-range and more abundant in the carboplatin resistant state.

### mTOR, WNT signaling, and other cancer pathways are associated with increased looping in carboplatin resistance

**Figure 7:**
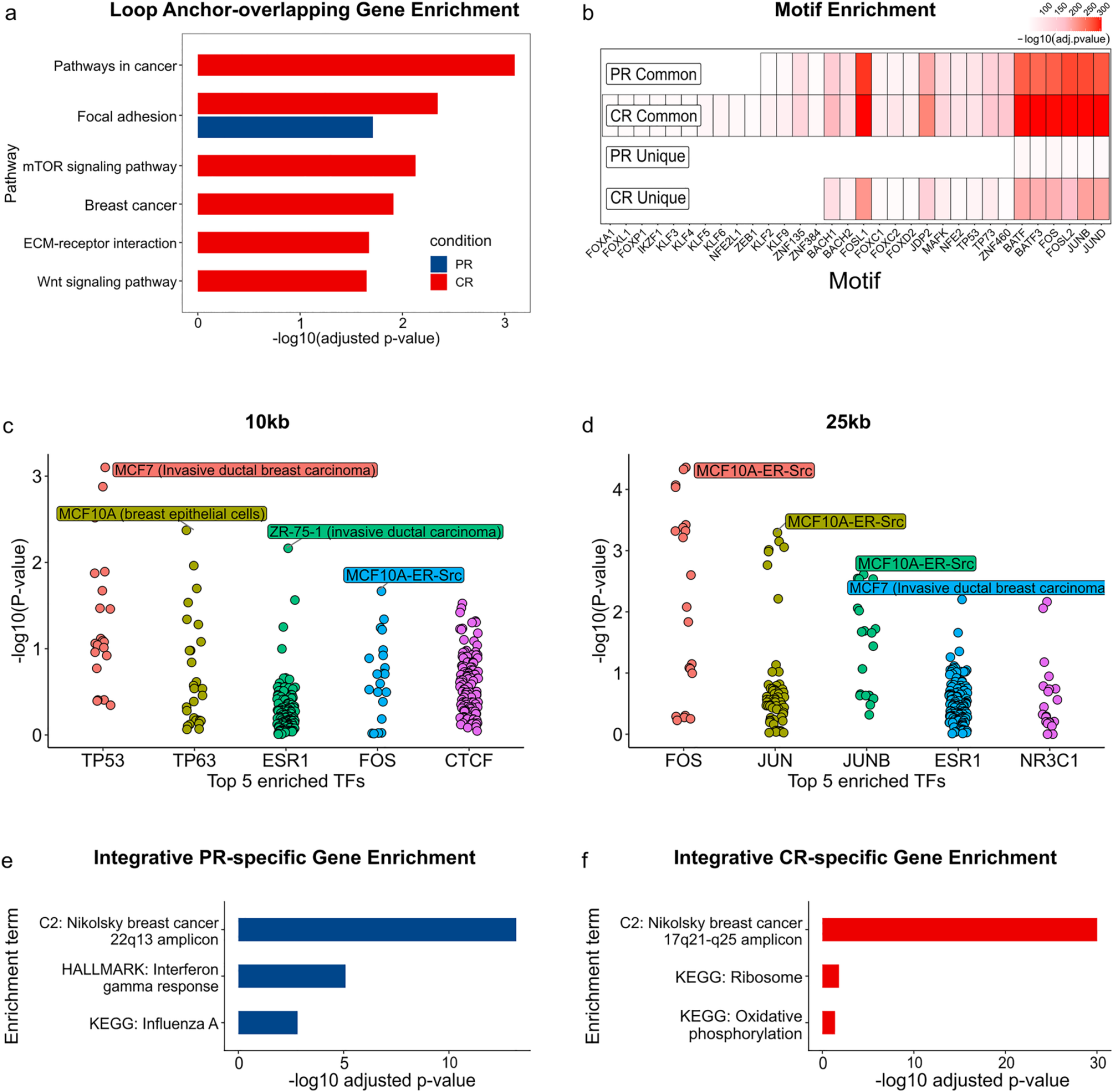
Functions and transcription factors enriched in condition-specific loop anchors. (**a**) Most significant hypergeometric enrichments of genes overlapping condition-specific loop anchors in KEGG pathways. Blue/red colors indicate PR/CR-specific enrichments. (**b**) MEME motif enrichment analysis at 10 kb resolution. Red gradient represents the enrichment level of transcription factor motifs (X-axis) in the condition-specific and common loop anchors (Y-axis). (**c**,**d**) Transcription factor enrichment analysis at 10 kb and 25 kb resolutions (UniBind). X-axis—transcription factors, Y-axis—−log10(enrichment *p* value), each dot represents cell/tissue-specific set of ChIP-seq peaks. (**e**,**f**) Most significant hypergeometric enrichments of genes supported by at least four pieces of evidence in the CR/PR condition. All analyses were performed on open chromatin regions (ATAC-seq) overlapping the condition-specific and common loop anchors.

To understand the functional significance of CR-specific interactome changes, we performed functional enrichment analysis of genes overlapping condition-specific loop anchors under the assumption that such anchors facilitate condition-specific enhancer-promoter interactions. Analysis of KEGG pathways identified genes overlapping common anchors being enriched in “Cell cycle” (25 genes, FDR = 9.44E−3) pathway (Supplementary Table S4). Genes overlapping PR-specific anchors were marginally enriched in the “Focal adhesion” KEGG pathway (23 genes, FDR = 1.94E−2); however, other genes from this pathway overlapped CR-specific anchors and were also enriched in it (39 genes, FDR = 4.52E−3). In contrast, genes overlapping CR-specific anchors were strongly enriched in “Pathways in cancer” (90 genes, FDR = 7.99E−4), “Breast cancer” (29 genes, FDR = 1.22E−2), and other cancer-specific pathways (“Gastric cancer”, “Glioma”, “Hepatocellular carcinoma”) (Fig. 7a). We also observed CR-specific enrichments in “mTOR signaling pathway” (31 genes, FDR = 7.45E−3), “ECM-receptor interaction” (18 genes, FDR = 2.12E−2), “Wnt signaling pathway” (29 genes, FDR = 2.24E−2), and similar cancer-related pathways. These results suggest that many cancer-related genes and pathways may be regulated by CR-specific loops.

### TP53, TP63, BATF, and FOS-JUN motifs are enriched in anchors of CR-specific loops

We hypothesized that carboplatin resistance-specific loops may enable regulation through interactions with specific transcription factor (TF) binding sites. Such transcription factors are commonly identified by motif enrichment analysis. Given the low resolution of Hi-C data (10 kb), we focused on open chromatin regions within the PR/CR-specific anchors. That is, anchors were intersected with the “Cancer/epithelial” ATAC-seq data from Meuleman et al.^41^, and only open chromatin regions were considered.

Using the AME tool from the MEME suite (Methods), we identified TP53, TP63, BATF, and FOS-JUN family of transcription factor motifs (JASPAR 2022) as significantly enriched in the CR-specific anchors (FDR < 1.00E−70, Fig. 7b, Supplementary Table S4). These motifs were also enriched in anchors common to PR and CR, suggesting that general features of loop anchors that are independent of carboplatin resistances may drive these enrichments. Therefore, we performed a discriminative analysis of CR/PR-specific versus corresponding CR/PR common anchors. We did not observe significant enrichments in the PR-specific versus PR-common anchors. In contrast, confirming the results of the shuffled analysis, FOSL1-JUND, BATF3, TP53, TP73, and other TFs were distinctly enriched in the CR-specific versus CR-common comparison (FDR = 2.23E−4/3.23E−4/7.30E−4/4.81E−3, respectively). These observations suggest TP53, TP63, BATF, and the FOS-JUN family of transcription factors may be regulating gene expression change via CR-specific looping.

We also performed enrichment analysis in cell-/tissue-specific experimentally obtained TFBSs from ChIP-seq experiments using UniBind^42^. Differential analysis of CR-specific versus PR-specific anchors at 10 kb resolution similarly identified TP53 and TP63 as the topmost enriched transcription factors, followed by ESR1 (hypergeometric *p* value = 7.94E−4/4.27E−3/6.91E−3, respectively) (Fig. 7c). Notably, these enrichments were breast cancer-specific (MCF7 (invasive ductal breast carcinoma), MCF10A (breast epithelial cells), ZR-75-1 (invasive ductal carcinoma)), reflecting the breast cancer origin of UCD52 cell line and a more aggressive phenotype in the CR condition. We also observed enrichment of FOS (*p* value = 2.16E−2, MCF10A-ER-Src experiment) and CTCF (*p* value = 3.00E−2); the latter is expected as we are focusing on loop anchors known to be enriched for CTCF binding. At 25 kb resolution, FOS, JUN, and JUNB from MCF10A cell line were the most enriched (*p* value = 4.36E−5/5.01E−4/2.45E−3) (Fig. 7d). These structurally and functionally related transcription factors are collectively described as activating protein-1 (AP-1), a multi-functional complex that mediates gene regulation during cell proliferation, differentiation and apoptosis or transformation, and tumorigenesis, and has long been known to be involved in multidrug resistance in breast cancer cells^43^. Together with the MEME analysis, the UniBind enrichments add FOS-JUN family of transcription factors to the list of potential regulators of carboplatin resistance.

### Smaller, stronger TADs are abundant in carboplatin resistance and enriched in cancer-related genes and pathways

Given the increased interaction strength and the number of loops at shorter distances, we expected to detect more TADs in the CR condition. Indeed, using Insulation Score (IS, see Methods), we detected more TADs in the CR condition (8152 vs. 5870 in PR, Supplementary Table S5). In contrast to Mustache loops that do overlap, IS detects consecutive TAD boundaries. Therefore, the larger number of TADs in the CR condition corresponded to the significantly smaller average TAD size (Median size 240 kb in PR vs. 190 kb in CR, Wilcoxon *p* value = 2.45E−46) (Supplementary Figure S6A). The number of CR-specific TADs was larger than that of PR-specific TADs (5437 vs. 3155), also reflected in the number of anchors (total and condition-specific). Similarly, the CR-specific TADs were smaller than the PR-specific TADs (Median size 360 kb vs. 210 kb, Wilcoxon *p* value = 4.25E−125). Considering size ranges, we observed fewer condition-specific TADs less than 100 kb and more TADs larger than 500 kb (Supplementary Figure S6B). Similar to the condition-specific loop anchors, the condition-specific TAD boundaries were depleted in deletions and duplications detected across both the PR and CR genomes (*p* value = 3.00E−3–1.00E−4). The aggregate peak and TAD analyses (APA, ATA) confirmed stronger looping and interactions within TADs in the CR condition (Supplementary Figure S6E,F). We investigated the enrichment of genes overlapping CR-specific and PR-specific TAD boundaries and found the “Pathways in cancer” KEGG pathway was among the most significant in CR (87 genes, FDR = 8.48E−4) while nothing was significant in PR. These results support previous observations that short-range interactions and loops strengthen in the carboplatin-resistant state and are associated with cancer-related genes.

### Integrative analysis suggests activation of mitochondrial metabolism and cancer-associated pathways/targets in carboplatin resistance

To prioritize genes affected in carboplatin resistance, we combined multiple layers of evidence into a CR-specific importance score. This score is a sum of evidence prioritizing genes significantly upregulated in CR (RNA-seq), amplified and overlapped CR-specific copy number variants (WGS), located in compartments switching into active state (dcHiC), and overlapped CR-specific loop anchors (Mustache) and TAD boundaries (hicFindTADs) (Supplementary Table S6). Examples include *MCUB*, a part of mitochondrial calcium uniporter complex known to play a role in chemoresistance^44^, *RPL38* ribosomal protein, a part of gene expression signature of cisplatin resistance^45^, *ABCA5*, ATP binding cassette subfamily A member 5, upregulation of which has been associated with poor outcome^36^, *IL17D*, an interleukin expression of which has been correlated with drug resistance^46^, and *BRCA1*, a part of DNA repair pathway associated with drug resistance^47^. We validated that *MCUB* and *BRCA1* had increased protein expression in carboplatin resistance (Supplementary Figure S7).

As expected, enrichment analysis on CR- and PR-specific genes supported by four or more of these criteria identified functional signatures previously observed with each omics data type separately (Fig. 7e,f). Paralleling observations from RNA-seq and dcHiC analyses, the CR-specific genes (regulation gained in CR) were enriched in “Ribosome” (19 genes, FDR = 1.52E−2), “Oxidative phosphorylation” (16 genes, FDR = 4.14E−2) KEGG pathways and similar gene ontologies and hallmark signatures (Supplementary Figure S8). Consistent with our observation of ABC transporter amplification on chr17, they were overrepresented among CR-specific genes and enriched in the “Nikolsky Breast Cancer 17q21-q25 Amplicon” signature (75 genes, FDR = 9.62E−31). Enrichment of the PR-specific genes (regulation lost in CR) expectedly showed immune-related functions detected as downregulated in CR, such as “Influenza A” KEGG pathway (27 genes, FDR = 1.54E−3), “Regulation of Viral Process” ontology (32 genes, FDR = 2.00E−5), and “Interferon Gamma Response” hallmark signature (43 genes, FDR = 8.16E−6) (Supplementary Table S6).

## Discussion

This study presents an integrative analysis of carboplatin resistance in breast cancer. The comparison of genomic (WGS), transcriptomic (RNA-seq) and 3D (Hi-C) datasets provides a unique perspective of mechanisms that promote acquired carboplatin resistance. Our study focuses on carboplatin resistance which is important as this drug has become an integral component of most TNBC treatment regimens. Second, we utilize a PDX model of TNBC as a better physiological experimental system for tumor development^48^, in contrast to cultured cell lines. Third, we integrated 3D interactome rewiring with genomic and transcriptomic changes and, in line with previous observations^47,49^, identified amplification of ABC transporters, mitochondrial energy metabolism, and several cancer-related pathways/genes/transcription factors as potential mechanisms of carboplatin resistance.

A limitation of our study is that it reports observations from one experimental model. Yet, our overlapping layers of evidence from multi-omics data provide a coherent picture of the 3D genome and transcriptome changes in the carboplatin-resistant state. Furthermore, many of our observations have been supported by other studies. For example, increase in short-range interaction frequencies and predominant switching of chromatin into a more active state in endocrine-resistant ER + breast cancer may reflect decompaction of constitutive heterochromatic regions, a known hallmark of carcinogenesis^50^. Differences in A/B compartment switching observed in the carboplatin resistant state support previously reported interaction strength increase in the A compartment with the parallel decrease in the B compartment under Decitabine treatment of endocrine-resistant breast cancer cell lines^50^. Paralleling our observations of the larger number of TADs in the CR condition, an increase in the number of TADs in B-cell lymphoma^51^ and prostate cancer^13^ has been reported. Furthermore, cancer TADs are known to be smaller than normal^13^, supporting our results. However, unlike our observations, loss of chromatin interactions was observed in Tamoxifen-resistant (but not in Fulvestrant-resistant) breast cancer cells^52^. Also, the development of endocrine resistance led to a significant increase in TAD size in Fulvestrant-resistant (but not in Tamoxifen-resistant) breast cancer cells^52^. These discrepancies can be attributed to different mechanisms driving endocrine- and carboplatin resistance, and differences in PDX mouse models. In summary, our results together with other studies warrant further work to refine the mechanisms of 3D genome remodeling in chemoresistance under different conditions.

Our findings of upregulated oxidative phosphorylation and ribosomal proteins suggest the role of mitochondrial energy metabolism in carboplatin resistance. Mitochondria generate metabolic energy by converting carbohydrates and fatty acids to ATP by glycolysis and oxidative phosphorylation (OXPHOS). Most mitochondrial proteins are translated on free cytosolic ribosomes and imported into the organelle by specific targeting signals. Some recent studies have shown that OXPHOS can be upregulated in certain cancers and suggest the role of *TP53*^53^, and OXPHOS inhibitors have shown promise in various tumors^54^. Interestingly, Lonidamine, a drug interfering with OXPHOS, has been known to potentiate the effect of chemotherapy on platinum-resistant ovarian cancer cells for over 25 years^55^. Furthermore, the OXPHOS pathway has recently been described as a mediator of chemotherapy resistance in TNBC^27^. Together with the emerging understanding of the role of mitochondrial components in chemotherapy resistance^44^, our results suggest that the mitochondrial metabolic processes may hold promise in overcoming carboplatin resistance.

Besides gene expression and structural variants, other layers of genomic information have been linked to 3D genome changes. DNA methylation has recently emerged as a major player in 3D chromatin remodeling in endocrine-resistant breast cancer^52^. Open chromatin (ATAC-seq) data and transcription factor binding profiles (e.g., CTCF ChIP-seq) are frequently used in the analysis. These (epi)genomic data are often obtained from public repositories for well-established cell- and tissue types. In contrast, our experimental system (UCD52 PDX model) is relatively unexplored and does not have readily available additional omics data. Therefore, generation of other genomic datasets, e.g., Smc1 or Rad21 ChIP-seq, to be integrated with our current data is of our future priorities. Furthermore, we performed our experiments on bulk tissue which obscures high heterogeneity of TNBC and warrants the use of single-cell technologies^56^. One rationale was the hypothesis that repeated carboplatin treatment will minimize heterogeneity by selecting resistant clones. This may explain the better agreement between Hi-C replicates in the CR condition (Fig. 4a). Our future plans include using single-cell technologies, including scHi-C, and integrating multi-omics data on a single-cell level.

## Conclusions

Our study is the first to provide a unique multi-omics dataset and integrative analysis of carboplatin-resistance in TNBC. It addresses several limitations of current studies. Specifically, we focused on TNBC as the most deadly and incurable subtype, instead of luminal subtypes with better survival prognosis and available treatment strategies. We utilized a PDX model as an experimental system better reflecting human tumor development. We investigated resistance to carboplatin, one of the platinum-based drugs, instead of better studied resistance to endocrine therapies. We took a multi-omics integrative approach by generating deeply sequenced Hi-C data, whole-genome sequencing, and RNA-seq. This approach allowed us to highlight the role of long noncoding RNAs and amplification of ABC transporters and prioritize mitochondrial metabolism and oxidative phosphorylation pathways as the possible mechanisms of carboplatin resistance.

## Methods

### Basal-like TNBC PDXs

The UCD52 basal-like triple-negative breast cancer PDX model was obtained from the University of Colorado. Tumors were grown in the mammary fat pads of female non-obese diabetic severe combined immunodeficient gamma (NSG) mice (The Jackson Laboratory, strain #005557). Tumors were allowed to grow to $\sim 70-100m{m}^{2}$ in size before being excised and prepared into a single-cell suspension using a previously described protocol^57^. Single-cell suspensions of PDX cells were used for serial passaging. Tumor cells were resuspended 1:1 in Matrigel (Corning) and injected into the right mammary gland of female NSG mice (500 K cells/injection). Single-cell suspensions were also used for Hi-C, WGS, and RNA-seq. All studies involving mice were approved by the Virginia Commonwealth University (VCU) Institutional Animal Care and Use Committee (IACUC) (Protocol# AD10001247), and all experiments were performed following IACUC guidelines and regulations. The results of animal experiments are reported in accordance with the ARRIVE guidelines 2.0.

### Development of a Carboplatin Resistant Subline

UCD52 tumors were allowed to grow in female NSG mice. Once tumors were $\sim 25-50m{m}^{2}$, mice were randomizet into two groups, one receiving three 40 mg/kg carboplatin doses administered intraperitoneally (treatment group) and another untreated (control group). Treatments were spaced four days apart. Treatment with carboplatin initially shrunk tumors and prevented tumor growth for a period of time. When tumor growth resumed, UCD52 tumors were harvested, prepped into single-cell suspensions, and serially passaged into new recipient mice. This process was repeated until the tumor was no longer responsive to carboplatin, yielding a derived carboplatin-resistant subline of UCD52. Mice bearing the carboplatin-resistant subline of UCD52 continued to receive the aforementioned regimen of carboplatin treatment to maintain the phenotype.

### Sample preparation

Hi-C sequencing was performed in replicates by Arima Genomics (San Diego, CA) as described^58^. WGS was performed by the Beijing Genomics Institute (BGI) according to the manufacturer’s protocol. RNA was prepared with the Qiagen RNeasy mini kit. Sequencing libraries were prepared with NEBNext Ultra II RNA Library Prep Kit for Illumina using manufacturer’s instructions (New England Biolabs). The sequencing libraries were multiplexed and clustered onto a flowcell. After clustering, the flowcell was loaded onto the Illumina HiSeq instrument according to the manufacturer’s instructions. The samples were sequenced using a 2 × 150 bp Paired End (PE) configuration. Image analysis and base calling were conducted by the HiSeq Control Software (HCS). Raw sequence data (.bcl files) generated from Illumina HiSeq was converted into fastq files and de-multiplexed using Illumina bcl2fastq 2.17 software. One mismatch was allowed for index sequence identification. Approximately 30 M reads were obtained per sample^59^.

### Hi-C

The data was processed using the Juicer v.1.6 pipeline^60^ and hg38 human genome assembly, which we showed to be the most optimal setting for PDX Hi-C data processing^58^. Reproducibility was estimated using the Python implementation of HiCRep^61^ at 1 Mb resolution. Multi-dimensional scaling and hierarchical clustering were performed on the matrix of pairwise chromosome-averaged HiCRep measures. Data resolution was estimated using the method introduced in Rao’s 2014 paper (the number of bins with > 1000 contacts should be at least 80% of the total number of bins, calculate_map_resolution.sh script)^5^. The maximum resolution per merged replicate (10 kb) was selected unless specified otherwise. Distance-dependent decay of interaction counts was obtained using HiCExplorer’s hicPlotDistVsCounts v.3.6 tool^62^, and the decay parameters were estimated using the poweRlaw v.0.70.6 R package^63^. Differential decay analysis was performed using the GENOVA v.1.0.0 R package^64^ on 100 kb-resolution data.

### A/B compartment analysis

To directly quantify the tendency of each region to interact with the other regions in either A or B compartments, we calculated several A/B compartment strength metrics. Briefly, .hic files were converted to multi-resolution .mcool files using the hic2cool tool v.0.8.3 (https://github.com/4dn-dcic/hic2cool). Data at 100 kb resolution was iteratively corrected and the AB compartment analysis was performed using the GENOVA v.1.0.0 R package. 100 kb bins were grouped into 50 percentile groups based on their PC1 (1st eigenvector) value. Within pairwise combinations of the 50 percentile groups, average contact enrichments (obs/exp) between bins were calculated, and log2 of the contact enrichment scores were plotted as a heatmap saddle plot. Summarized A-A and B-B compartment strengths were calculated as the mean log2 contact enrichment between the top (A-A) or bottom (B-B) 20% of PC1 percentiles and between the top and bottom 20% of PC1 percentiles for A-B compartment strength, excluding chrY and chrM. The compartmentalization score was calculated as previously described using mean contact enrichments for A-A, B-B, and A-B following this formula: $log((A - A){*(}B - B{)}/(A - B)^{2} )$^65^.dcHiC (differential compartment analysis of Hi-C) is a method that identifies statistically significant differences in compartmentalization among two or more contact maps, including changes that are not accompanied by a compartment flip^39^. dcHiC first employs a time- and memory-efficient R implementation of singular value decomposition (SVD) to achieve the eigenvalue decomposition of each Hi-C contact map. This is followed by automated selection to find the principal component and its sign (reoriented if needed) that best correlates with gene density and GC content per sample. The resulting compartment scores are then quantile normalized, and a multivariate score (Mahalanobis distance) is computed to detect the outliers. The multivariate score is then used for computing the final statistical significance (Chi-square test) of differences in compartmentalization. dcHiC was applied to PR and CR Hi-C data at 250 kb resolution with default parameters. Chromosome-wise principal compartments for each sample were selected based on what best correlates with gene density and GC content. The compartment scores and signs were further manually inspected to refine the assignment (Supplementary Table S3). Statistically significant differences were called at FDR = 0.3.

### Loop analysis

Mustache v1.2.4^4^ was used to detect significant intrachromosomal loops in reads from the CR and PR strains. Merged replicate Hi-C maps of 10 kb resolution were inputted into Mustache and analyzed using VC normalization with a *p* value threshold of 0.1. The output calls were filtered for significance at FDR < 0.05.

A loop is defined by two anchors forming an interaction pair. Loops called in PR and CR conditions were separated into condition-specific and common loops using the GenomicInteractions v.1.28.0 R package^66^. Loops were considered common allowing for a resolution-size flank when considering overlaps. Similarly, anchors within a resolution-size flank were considered overlapping. Anchors were separated into PR/CR-specific and common anchors using the GenomicRanges v.1.46.1 R package^67^. Loops and anchors overlapping centromeres, telomeres, and excludable regions (the excluderanges v.0.99.6 R package, https://github.com/dozmorovlab/excluderanges) were removed. TNBC-specific enhancers were obtained from Huang et al.^68^. The association (enrichment) analysis was performed using the regioneR v.1.30.0 R package.

### Motif analysis

Open chromatin regions within the PR/CR-specific and common anchors were analyzed using the MEME suite via the memes v.1.2.0 R package^69^. The AME analysis identifies enriched motifs in a set of target sequences as compared with shuffled sequences. Additionally, AME discriminative analysis compares sets of sequences for motifs enriched in one of the sets. The UniBind^42^ differential enrichment analysis was run with default settings. Enrichment results supported by at least 50 open chromatin regions and two ChIP-seq experiments were kept.

### CTCF overlap analysis

The proportion of PR/CR-specific and common anchors overlapping CTCF motifs were quantified using GenomicRanges v.1.46.1 R package^67^. Anchors were reduced to merge overlapping sets prior to counting the number of CTCF motifs overlapping each anchor. The anchors overlapping multiple or single CTCF sites were extracted and quantified, and anchors overlapping no CTCF sites were flanked in both directions by one bin equal to Hi-C resolution. The overlap between the extended anchors and CTCF motifs was calculated, and the anchors were classified as containing at least one (overlapping) or no CTCF sites.

### RNA-seq analysis

A custom reference genome was created consisting of human, mouse, and viral genomes. The GRCh38.d1.vd1 Reference Sequence of the human genome, which includes viral genomes, and the corresponding annotation file were obtained from NCI’s Genomic Data Commons. It was merged with the GRCm38 M12 Gencode release of the primary assembly mouse genome and its corresponding annotation file. Chromosomes were labeled with organism-specific prefixes before concatenation, similar to the method described by Callari et al.^70^.

FastQC v.0.11.8 and MultiQC v.1.8 were used to perform initial quality control on FASTQ files. Reads were trimmed to remove adaptors and poor quality bases using Cutadapt v.1.15^71^. STAR v2.5.2b^72^ was used to index the concatenated reference genome and perform the alignment. The FASTQ files were aligned to the concatenated genome with the following STAR parameter settings: –outSAMtype BAM Unsorted –outSAMorder Paired –outReadsUnmapped Fastx –quantMode TranscriptomeSAM –outFilterMultimapNmax 1. Read counting to obtain gene expression values from the aligned BAM files utilized a transcript reference file created from the concatenated genome and was performed using the Salmon v0.8.2 “quant” algorithm with the library type set to “IU”^73^. The counts were imported into R v.4.0.3 using the tximport R package v.1.18.0^74^. Differential expression was performed using the edgeR v.3.36.0 R package^75^ on human transcript counts.

### Functional enrichment analysis

Functional enrichment analysis (GO, KEGG) using a hypergeometric test was performed using the enrichr R package v.3.0^76^. Pre-ranked GSEA was performed with 1000 permutations on gene sets obtained from the Molecular Signature Database hosted at the Broad Institute (MSigDB) (msigdbr v.7.4.1). For gene expression differences, genes were ranked by −log10(p-value), with the sign reflecting the directionality of fold change. For gene coverage differences, genes were ranked by log2 coverage differences weighted (multiplied) by the average coverage for the corresponding gene. This was done to prioritize genes with high average coverage and high coverage differences. For AB compartment switches, genes were ranked by eigenvector differences. All statistical calculations were performed in R v4.1.0 and Bioconductor v.3.14.

### Expression correlation with other measures

Gene expression changes were compared with WGS coverage changes and AB compartment differences (Eigenvector differences). Gene expression coverage counts were obtained with htseq-count v.0.11.1 using “–order = pos” and “–stranded = no” settings^77^ and Gencode v.39 gene annotations. Genes with small changes in either measure (absolute log2FC less than 1 SD of the corresponding distribution) were removed.

### WGS structural variant (SV) analysis

Mouse reads were removed from PDX WGS data by aligning the data to the combined hg38 and mm10 genome and retaining reads aligned to the human portion, as recommended^70^. Paired-end reads were aligned to the hg38 human genome assembly using bwa mem v.0.7.17^78^, duplicates were removed using picard MarkDuplicates v.2.18.29^79^. Sorting, indexing, alignment statistics extraction was performed using samtools v.1.7^80^. Coverage bigWig files at 10 kb resolution were obtained with the deeptools’s v.3.5.0^81^ bamCoverage tool. Coverage differences (log2 ratio) were obtained using deeptools bamCompare. The DNAcopy v.1.68.0 R package^82^ was used to segment coverage differences using the Circular Binary Segmentation algorithm.

We predicted SV using three different tools: delly v.0.1.19^83^, lumpy v.0.2.13^84^, and breakdancer v.1.1.12^85^, and filtered out the SVs calls with less than 5 PE reads of evidence to increase call stringency. We then defined a consensus call set by overlapping the results with bedtools v2.30.0^86^, with a window size of 1kbp for translocations and 500 bp for all other SV types. To reduce the number of false positives, we kept only the SVs that were recovered by at least two algorithms. Finally, we intersected these consensus calls to identify sample-specific variants.

### Immunohistochemical Staining of Formalin-Fixed Paraffin-Embedded PDX Sections

Immunohistochemistry (IHC) was performed on formalin fixed paraffin embedded tissue sections using rabbit primary anti-BRCA1 (HPA057371) or anti-MCUB (HPA048776) antibodies from Sigma-Aldrich, (St. Louis MO, USA), as well as polymer/HRP secondary antibody with 3,3’-Diaminobenzidine (DAB + chromogen and DAB + substrate buffer (K4011), Dako (Carpinteria CA, USA). Tissues were counterstained with hematoxylin (GHS332, Sigma-Aldrich), coverslips were mounted with Permount (SP15, Fisher) and slides imaged at 400X magnification with a Carl Zeiss™ AxioLab™ A1 light microscope and processed with ZEN Digital Imaging for Light Microscopy software.

### Protein quantification of BRCA1 and MCUB

IHC images were quantified in ImageJ^87^ (two replicates per condition * three areas = 6 measurements). The Colour Deconvolution option was selected, and the stain H DAB was selected. Upon deconvolution, the Colour_2 image (DAB) was analyzed via Set Measurements for mean grey value only as the display value. The image was measured (Ctrl + M) to obtain a mean intensity value. The optical density (OD) was calculated as $OD = log_{10} \frac{255}{{meanintensity}}$. The differences in ODs were quantified using two-tailed t-test.

## Supplementary Information

Supplementary Table S1.

Supplementary Table S2.

Supplementary Table S3.

Supplementary Table S4.

Supplementary Table S5.

Supplementary Table S6.

Supplementary Legends.

Supplementary Figures.

## Data Availability

All raw and processed sequencing data generated in this study have been submitted to the NCBI Gene Expression Omnibus (GEO; https://www.ncbi.nlm.nih.gov/geo/), under accession number GSE201435. The whole genome sequencing data generated in this study have been submitted to the NCBI SRA database (https://www.ncbi.nlm.nih.gov/bioproject/), under accession number PRJNA832117: https://dataview.ncbi.nlm.nih.gov/object/PRJNA832117. All original code has been deposited at Github (https://github.com/dozmorovlab/PDXHiC_supplemental).
