# Publisher Correction: Rewiring of the 3D genome during acquisition of carboplatin resistance in a triple-negative breast cancer patient-derived xenograft

**DOI:** 10.1038/s41598-023-33284-y

**Published:** 2023-05-09

**Authors:** Mikhail G. Dozmorov, Maggie A. Marshall, Narmeen S. Rashid, Jacqueline M. Grible, Aaron Valentine, Amy L. Olex, Kavita Murthy, Abhijit Chakraborty, Joaquin Reyna, Daniela Salgado Figueroa, Laura Hinojosa-Gonzalez, Erika Da-Inn Lee, Brittany A. Baur, Sushmita Roy, Ferhat Ay, J. Chuck Harrell

**Affiliations:** 1 Department of Biostatistics, Virginia Commonwealth University, Richmond, VA 23298 USA; 2 Department of Pathology, Virginia Commonwealth University, Richmond, VA 23284 USA; 3 Department of Biology, University of Richmond, Richmond, VA 23173 USA; 4 Department of Biochemistry, Virginia Commonwealth University, Richmond, VA 23284 USA; 5 C. Kenneth and Dianne Wright Center for Clinical and Translational Research, Virginia Commonwealth University, Richmond, VA 23298 USA; 6 Center for Cancer Immunotherapy and Autoimmunity, La Jolla Institute for Immunology, La Jolla, CA 92037 USA; 7 Department of Pediatrics, UC San Diego—School of Medicine, La Jolla, CA 92093 USA; 8 Wisconsin Institute for Discovery, University of Wisconsin-Madison, Madison, WI 53715 USA; 9 Department of Biostatistics and Medical Informatics, University of Wisconsin-Madison, Madison, WI 53792 USA

Correction to: *Scientific Reports* https://doi.org/10.1038/s41598-023-32568-7, published online 03 April 2023

The original version of this Article contained an error in the Data availability section, where the GEO accession number was omitted.

“All raw and processed sequencing data generated in this study have been submitted to the NCBI Gene Expression Omnibus (GEO; https://www.ncbi.nlm.nih.gov/geo/). The whole genome sequencing data generated in this study have been submitted to the NCBI SRA database (https://www.ncbi.nlm.nih.gov/bioproject/) under accession number PRJNA832117, reviewer's accession link: https://dataview.ncbi.nlm.nih.gov/object/PRJNA832117. All original code has been deposited at Github (https://github.com/dozmorovlab/PDXHiC_supplemental).”

now reads:

“All raw and processed sequencing data generated in this study have been submitted to the NCBI Gene Expression Omnibus (GEO; https://www.ncbi.nlm.nih.gov/geo/), under accession number GSE201435. The whole genome sequencing data generated in this study have been submitted to the NCBI SRA database (https://www.ncbi.nlm.nih.gov/bioproject/), under accession number PRJNA832117: https://dataview.ncbi.nlm.nih.gov/object/PRJNA832117. All original code has been deposited at Github (https://github.com/dozmorovlab/PDXHiC_supplemental).”

The original Article has been corrected.

