# Author Correction: Rewiring of the 3D genome during acquisition of carboplatin resistance in a triple-negative breast cancer patient-derived xenograft

**DOI:** 10.1038/s41598-024-82374-y

**Published:** 2024-12-17

**Authors:** Mikhail G. Dozmorov, Maggie A. Marshall, Narmeen S. Rashid, Jacqueline M. Grible, Aaron Valentine, Amy L. Olex, Kavita Murthy, Abhijit Chakraborty, Joaquin Reyna, Daniela Salgado Figueroa, Laura Hinojosa-Gonzalez, Erika Da-Inn Lee, Brittany A. Baur, Sushmita Roy, Ferhat Ay, J. Chuck Harrell

**Affiliations:** 1 Department of Biostatistics, Virginia Commonwealth University, Richmond, VA 23298 USA; 2 Department of Pathology, Virginia Commonwealth University, Richmond, VA 23284 USA; 3 Department of Biology, University of Richmond, Richmond, VA 23173 USA; 4 Department of Biochemistry, Virginia Commonwealth University, Richmond, VA 23284 USA; 5 C. Kenneth and Dianne Wright Center for Clinical and Translational Research, Virginia Commonwealth University, Richmond, VA 23298 USA; 6 Center for Cancer Immunotherapy and Autoimmunity, La Jolla Institute for Immunology, La Jolla, CA 92037 USA; 7 Department of Pediatrics, UC San Diego—School of Medicine, La Jolla, CA 92093 USA; 8 Wisconsin Institute for Discovery, University of Wisconsin-Madison, Madison, WI 53715 USA; 9 Department of Biostatistics and Medical Informatics, University of Wisconsin-Madison, Madison, WI 53792 USA

Correction to: *Scientific Reports* 10.1038/s41598-023-32568-7, published online 03 April 2023

The original version of this Article contained an error in the Acknowledgements section.

 “This work was supported by the PhRMA Foundation Research Informatics Award, the George and Lavinia Blick Research Scholarship to MD, the NIH/NCI (1R01CA246182-01A1) grant, and the Susan G. Komen Foundation (CCR19608826) award to JCH, and NIH/NIGMS (R35-GM128938) grant to FA. Services and products in support of the research project were generated by the Virginia Commonwealth University Cancer Mouse Models Core Laboratory, supported, in part, with funding to the Massey Cancer Center from NIH-NCI Cancer Center Support Grant P30 CA016059.”

now reads:

“This work was supported by the PhRMA Foundation Research Informatics Award, the George and Lavinia Blick Research Scholarship to MD, the NIH/NCI (1R01CA246182-01A1) grant, and the Susan G. Komen Foundation (CCR19608826) award to JCH, NIH/NIGMS (R35-GM128938) grant to FA, and R01HG010045 to SR. Services and products in support of the research project were generated by the Virginia Commonwealth University Cancer Mouse Models Core Laboratory, supported, in part, with funding to the Massey Cancer Center from NIH-NCI Cancer Center Support Grant P30 CA016059.”

The original Article has been corrected.

