# Supplementary material for: Rewiring of the 3D genome during acquisition of carboplatin resistance in a triple-negative breast cancer patient-derived xenograft
Source: Sci Rep. 2023 Apr 3;13:5420. doi: 10.1038/s41598-023-32568-7 (PMC10070455; doi:10.1038/s41598-023-32568-7)
Supplement: Supplementary file 7 — Supplementary Legends. [file 41598_2023_32568_MOESM7_ESM.docx]

**Table S1. Transcripts differentially expressed in the CR condition and their functional significance.** “DEGs” sheet - differentially expressed transcripts at FDR < 0.1. “genes”, “biotype” “description” - gene annotations; “logFC” - log fold change; “logCPM” - average Counts Per Million, log2; “LR” - log-likelihood; “PValue”/“FDR” - non-/FDR-adjusted p-value. Bolded are downregulated genes overlapping TNBC-specific genes in^1^. “Summary” sheet - counts of up-and downregulated transcript types and the corresponding Chi-square p-value. “GSEA.” sheets - enrichment analysis of protein-coding genes in KEGG pathways, C2: curated gene sets, C5: ontology gene sets, and H: hallmark gene sets from MSigDb. “ID”, “Description” - functional signature, “NES” - normalized enrichment score (negative - enrichment in downregulated genes). “LncSEA.” sheets - enrichment analysis of non-protein-coding genes. “Direction” - whether up- (“UP”) or downregulated (“DN”) sets of lncRNAs were analyzed.

**Table S2. WGS coverage QC, summary of large deletions and duplications, gene coverage.** “QC” sheet - “samtools stats” alignment statistics, “log2FC” - metric log2 fold change between CR and PR conditions. Summary” sheet - chromosome-specific counts and width of deletions (DEL) and duplications (DUP) identified by the Circular Binary Segmentation algorithm on the log2 coverage ratio between the CR and PR conditions. “Genes” sheet - genes overlapping large deletions and duplications. “Coverage.RNAseq” sheet - Gene coverage and expression differences between the CR and PR conditions. “ensgene”/“symbol”/“biotype”/“description” - gene information, “PR”/“CR” - number of reds per gene in the corresponding condition, “Length” - gene length, “Average” - average coverage, “Coverage log2FC” - log2FC coverage difference between the CR and PR conditions, “Differential expression log2FC” - log2FC gene expression difference between the CR and PR conditions, “logCPM” - log2 counts per million average gene expression, “LR”/“PValue”/“FDR” - log-ratio, p-value, and FDR-corrected p-value. “Enrichr.” sheets - hypergeometric enrichment analysis of genes located in deleted or duplicated regions. Each worksheet corresponds to enrichment of “DEL” or “DUP” genes in corresponding MSigDb collections. “ID”, “Description” - signature description; “pvalue”, “p.adjust” - raw and FDR-corrected p-values; “Count”, “geneID” - number and list of genes enriched in a given signature; “Direction” - “DEL” or “DUP” regions associated with enriched genes. “GSEA.” sheets - GSEA enrichment results. “ID”, “Description” - functional signature, “NES” - normalized enrichment score.

**Table S3. Quality metrics and chromatin state switching analysis results (dcHiC).** “HiC.QC” sheet - Quality metrics of replicates and merged samples from the Juicer pipeline. “dcHiC.eigenvectors” sheet - Chromosome-specific eigenvectors. Correlation of principal components with with GC content (“GC.cor”) and transcription start sites (“TSS.cor”) were summarized into a “total.score”. “Genes.AA/BB/BA/AB” sheets - Genes overlapping regions switching chromatin state (FDR < 0.1), “D.EV” - eigenvector difference, negative corresponds to A-B switch, “COSMIC”, “BushmanLab”, “PID_C”, “PID_D” - indicator columns highlighting oncogenes annotated in COSMIC database^2^, cancer-related genes downloaded from Bushman lab (<http://www.bushmanlab.org/assets/doc/allOnco_May2018.tsv>), Pathway Implicated Driver (PID) genes with coding variants (PID-C) and noncoding variants (PID-N)^3^. “GSEA.XX” sheets - GSEA analysis of genes ranked by the magnitude and directionality of chromatin state change. KEGG pathways, C2: curated gene sets, C5: ontology gene sets, and H: hallmark gene sets MSigDb results are shown in the corresponding worksheets. “ID”, “Description” - gene signature, NES - normalized enrichment score (negative - enrichment in A-B switching genes).

**Table S4. Summary statistics and loop overlap analysis results (Mustache).** “Mustache.QC” sheet - Counts, width, and CTCF overlap summary statistics for the condition-specific and common loops. “Genes.PRunique/PRcommon/CRunique/CRcommon” sheets - Genes overlapping loop anchors unique or common for each condition. “CRunique/CRcommon/PRunique/PRcommon” sheets - hypergeometric enrichment analysis of genes overlapping condition-specific anchors in KEGG pathways. “Term” - gene set name, “Overlap” - the number of genes out of the set total, “P.value” and “Adjusted.P.value” - hypergeometric raw and FDR-corrected p-values, “Genes” - genes enriched in the set. “MEME.XX” sheets - MEME motif enrichment results. “Shuffled” - target regions vs. randomly shuffled regions, “Common” - condition-specific regions vs. common regions. A detailed output description is available at <https://meme-suite.org/meme/doc/ame-output-format.html>. “Unibind.10kb/25kb” sheets - Transcription factor binding sites enriched in the CR-specific loop anchors as compared with the PR-specific anchors, at 10kb and 25kb resolution. A detailed output description is available at <http://code.databio.org/LOLA/>.

**Table S5. Summary statistics and TAD boundary overlap analysis results (hicFindTADs).** “hicFindTADs.QC” sheet - Counts and width summary statistics for the condition-specific and common loops. “Genes.PRunique/PRcommon/CRunique/CRcommon” sheets - Genes overlapping TAD boundaries unique or common for each condition. “CRunique/CRcommon/PRunique/PRcommon” sheets - hypergeometric enrichment analysis of genes overlapping condition-specific boundaries in KEGG pathways. “Term” - gene set name, “Overlap” - the number of genes out of the set total, “P.value” and “Adjusted.P.value” - hypergeometric raw and FDR-corrected p-values, “Genes” - genes enriched in the set.

**Table S6. Multi-omics characterization of drug resistance-associated genes.** “Combined” sheet - drug resistance-associated genes supported by multiple layers of evidence. “CR.hot” and “PR.hot” - the sum of evidence from RNA-seq, WGS, Mustache, hicFindTADs, dcHiC, for either CR or PR conditions. “RNAseq.logFC”, “RNAseq.significant” - expression log2FC and an indicator whether a gene is significantly differentially expressed; “WGS.logFC”, “WGS.significant” - copy number log2FC and an indicator whether a gene is overlapping condition-specific copy number region; “Mustache.significant”, “hicFindTADs.significant” - indicators whether a gene overlaps CR-specific loop/TAD boundaries; “dcHiC.logFC”, “dcHiC.significant” - eigenvector log2FC and an indicator of chromatin state change (from PR to CR, e.g., BA means switching from inactive to active state). Significantly upregulated, amplified in CR genes, overlapping with CR-specific loop/TAD boundaries, and located in significant “BA” or “AA” chromatin state switching regions are prioritized for the CR condition and vice versa for the PR condition. Additional annotations include COSMIC database, cancer genes from Bushman lab, Pathway Implicated Drivers^3^, and TNBC enhancers^4^. “CR/PR.KEGG/C2/C5/H” sheets - Functional enrichment of genes supported by at least four pieces of evidence in the CR/PR condition in “KEGG” pathways, “H” hallmark gene sets, and other MSigDB collections (<http://www.gsea-msigdb.org/gsea/msigdb/collections.jsp>). “Term” - gene set name, “Overlap” - the number of genes out of the set total, “P.value” and “Adjusted.P.value” - hypergeometric raw and FDR-corrected p-values, “Genes” - genes enriched in the set.

1. Komatsu, M. *et al.* [Molecular features of triple negative breast cancer cells by genome-wide gene expression profiling analysis](https://doi.org/10.3892/ijo.2012.1744). *Int J Oncol* **42**, 478–506 (2013).

2. Forbes, S. A. *et al.* [COSMIC: Somatic cancer genetics at high-resolution](https://doi.org/10.1093/nar/gkw1121). *Nucleic Acids Res* **45**, D777–D783 (2017).

3. Reyna, M. A. *et al.* [Pathway and network analysis of more than 2500 whole cancer genomes](https://doi.org/10.1038/s41467-020-14367-0). *Nat Commun* **11**, 729 (2020).

4. Huang, H. *et al.* [Defining super-enhancer landscape in triple-negative breast cancer by multiomic profiling](https://doi.org/10.1038/s41467-021-22445-0). *Nat Commun* **12**, 2242 (2021).
