# Supplementary material for: Rewiring of the 3D genome during acquisition of carboplatin resistance in a triple-negative breast cancer patient-derived xenograft
Source: Sci Rep. 2023 Apr 3;13:5420. doi: 10.1038/s41598-023-32568-7 (PMC10070455; doi:10.1038/s41598-023-32568-7)
Supplement: Supplementary file 8 — Supplementary Figures. [file 41598_2023_32568_MOESM8_ESM.docx]

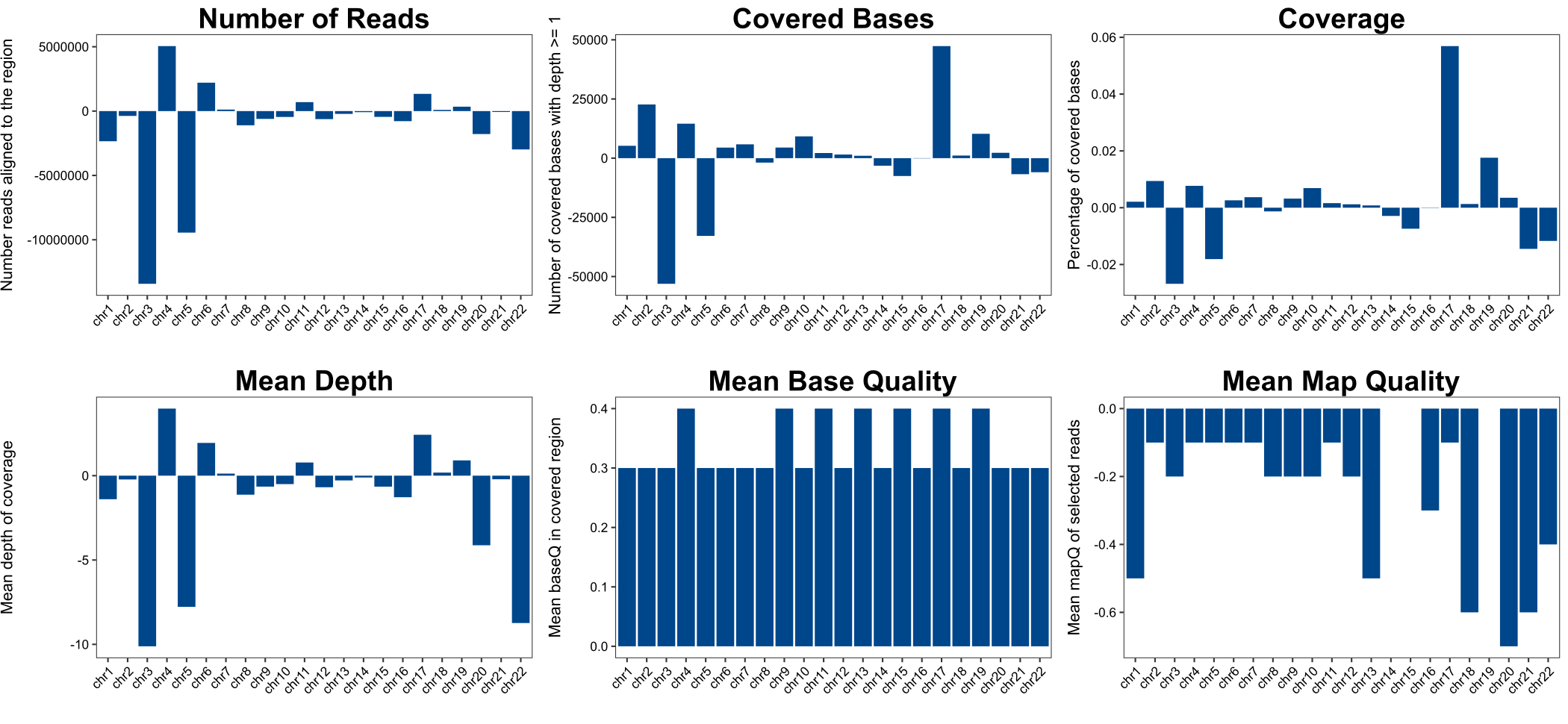


**Figure S1. WGS coverage statistic differences between UCD52CR and UCD52PR conditions.** The difference for each coverage metric outputted by samtools coverage (<https://www.htslib.org/doc/samtools-coverage.html>) is summarized in the corresponding plots. “Number of Reads” - number of reads that aligned to the region after filtering, “Covered Bases” - bases covered with a depth of at least one, “Coverage” - the proportion of bases covered, “Mean Depth” - mean coverage depth, “Mean Base Quality” - mean base quality for the region covered, “Mean Mapping Quality” - mean mapping quality of reads.


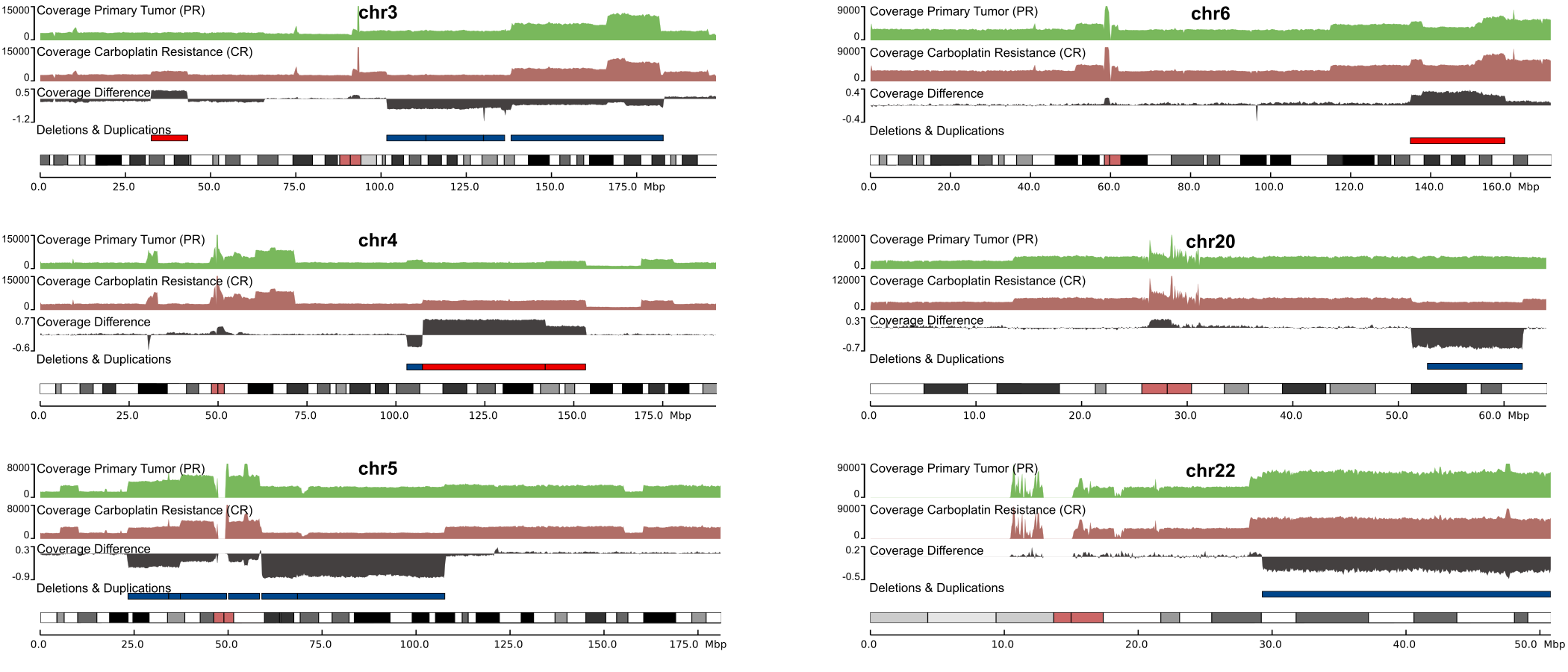
 **Figure S2. Coverage of selected chromosomes containing duplicated or deleted regions.** Coverage tracks for UCD52PR and UCD52CR conditions and the log2 fold change in coverage between the two conditions were plotted using pyGenomeTracks (<https://pygenometracks.readthedocs.io/en/latest/content/usage.html#pygenometracks>). Duplicated/deleter regions are shown in red/blue, respectively. Chromosome ideograms were generated with CoolBox (<https://gangcaolab.github.io/CoolBox/api.html>).


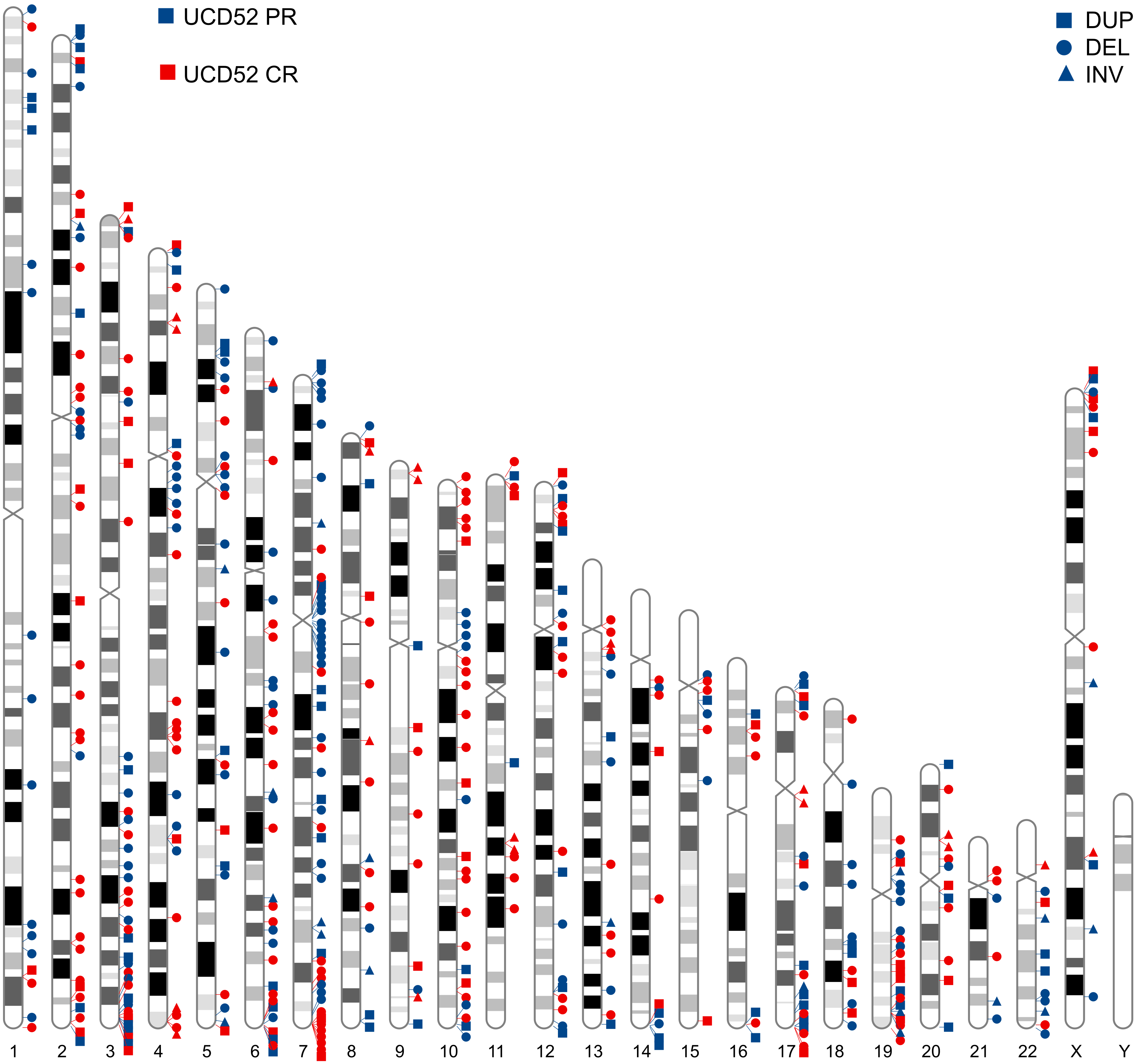
 **Figure S3. Whole genome karyotype with condition-specific deletions, duplications, and inversions detected by three SV callers (delly, lumpy, breakdancer).** Blue/red color indicate the UCD52 PR/CR conditions, box/circle/triangle indicate duplications, deletions, and inversions, respectively. Chromosome karyoplot was generated using the RIdeogram v.0.2.2 R package.


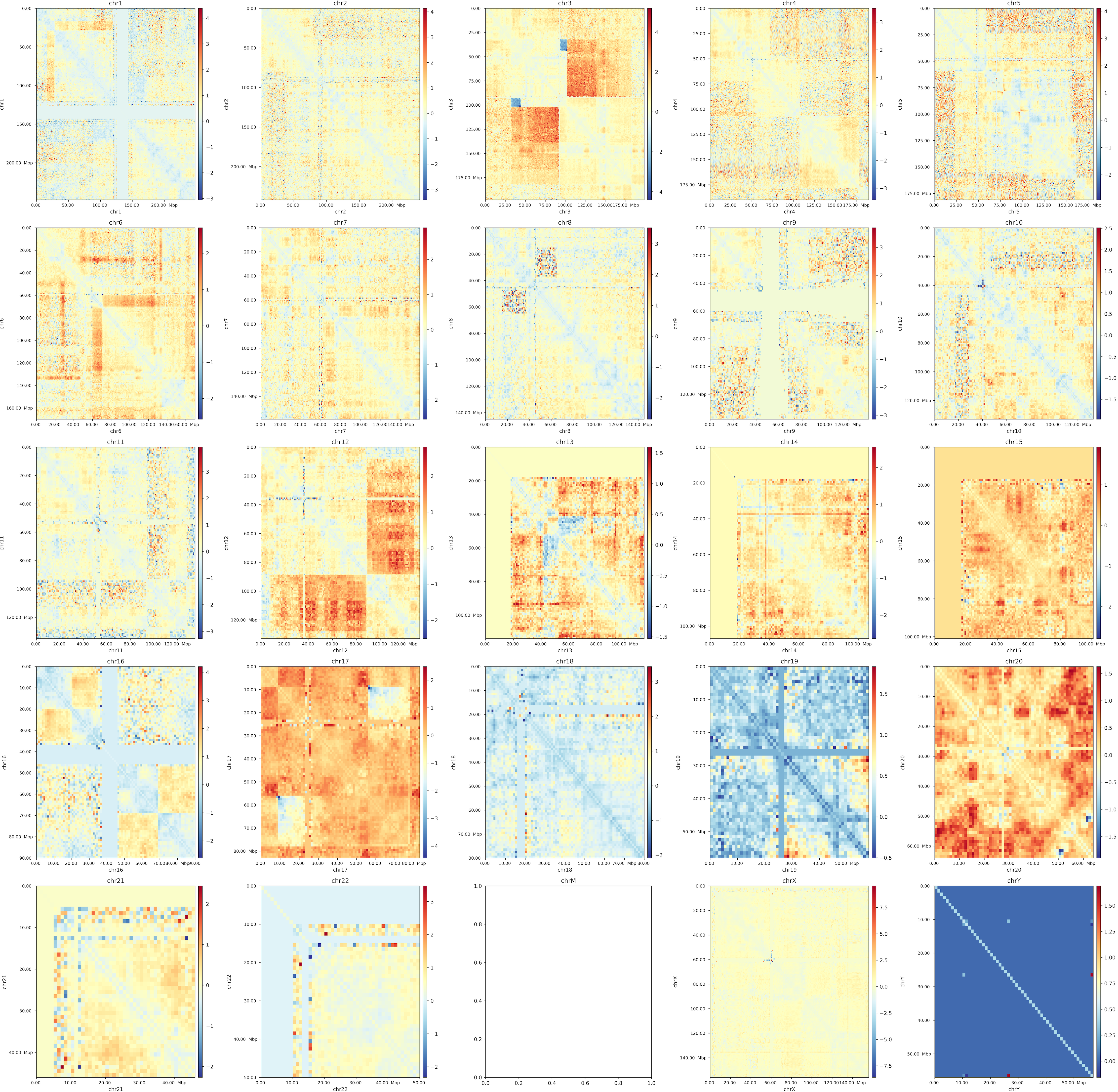
 **Figure S4. Differential chromosome-specific contact maps showing the CR vs. PR differences.** Blue-yellow-red gradient shows decrease-to-increase frequency of chromatin interactions.


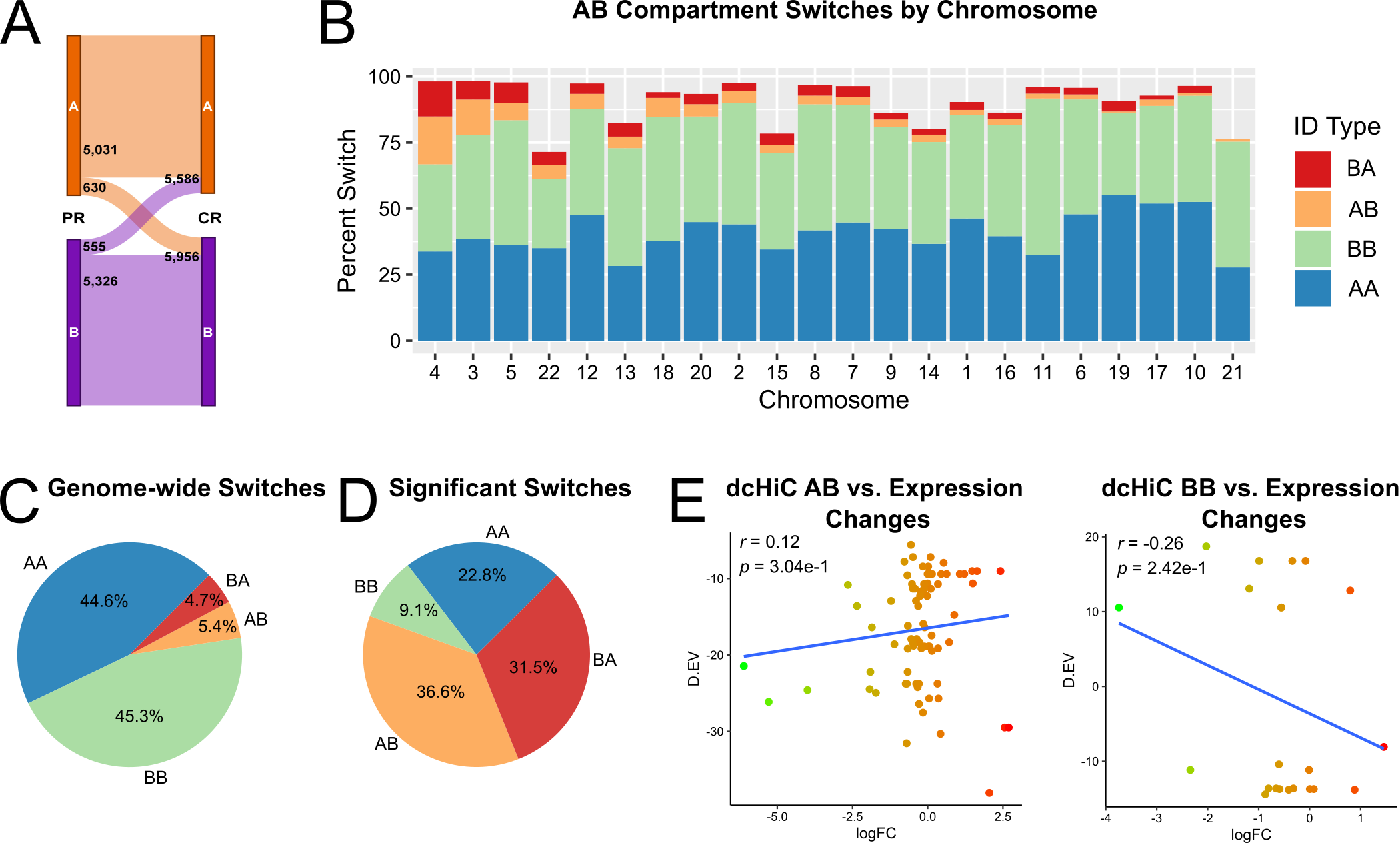
 **Figure S5. Chromatin state changes in drug resistance.** (A) Genomewide flow chart and (B) chromosome-specific proportions of the genome switching states between active A and inactive B compartments in the CR vs. PR comparison at FDR = 1. (C) Proportions of chromatin switches at FDR = 1 and (D) FDR < 0.3. (E) Correlation between gene expression- (X-axis) and chromatin state (eigenvector) changes (Y-axis) in AB and BB compartment switches.


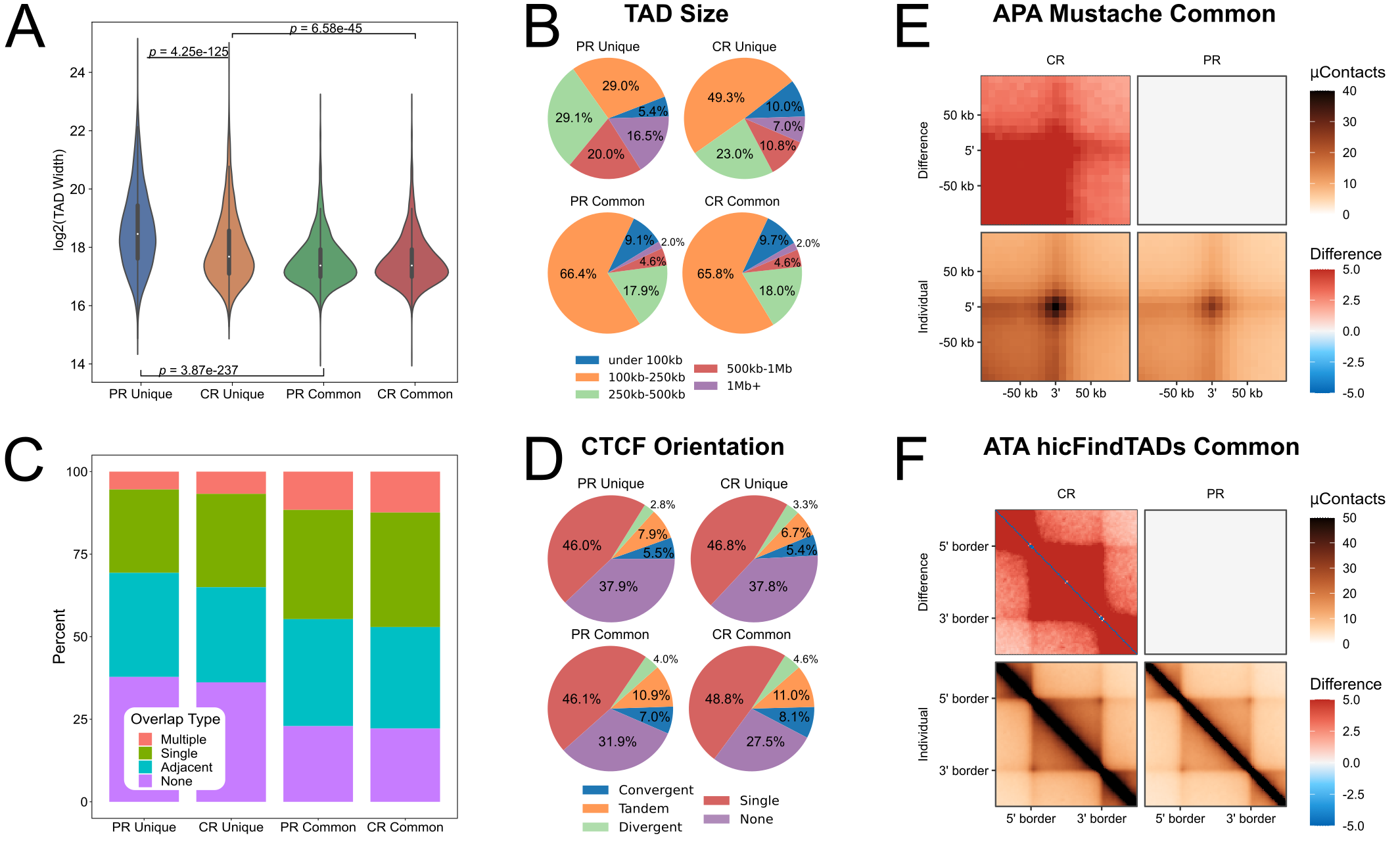
 **Figure S6. Width distribution and CTCF orientation at the condition-specific and common TADs.** (A) log2 width distribution violin plots and Wilcoxon p-values, (B) Size range comparison of TAD width distributions, (C) Proportions of TAD boundaries overlapping multiple, single, adjacent, or none CTCF binding motifs, (D) Proportions of TADs with various CTCF configurations at boundaries. (E) Differential Aggregate Peak Analysis (APA) and Aggregate TAD Analysis (ATA) of common loops and TADs. Brown color gradient indicates interaction strength, and the blue-to-red gradient indicates differences between two APA/ATA plots.


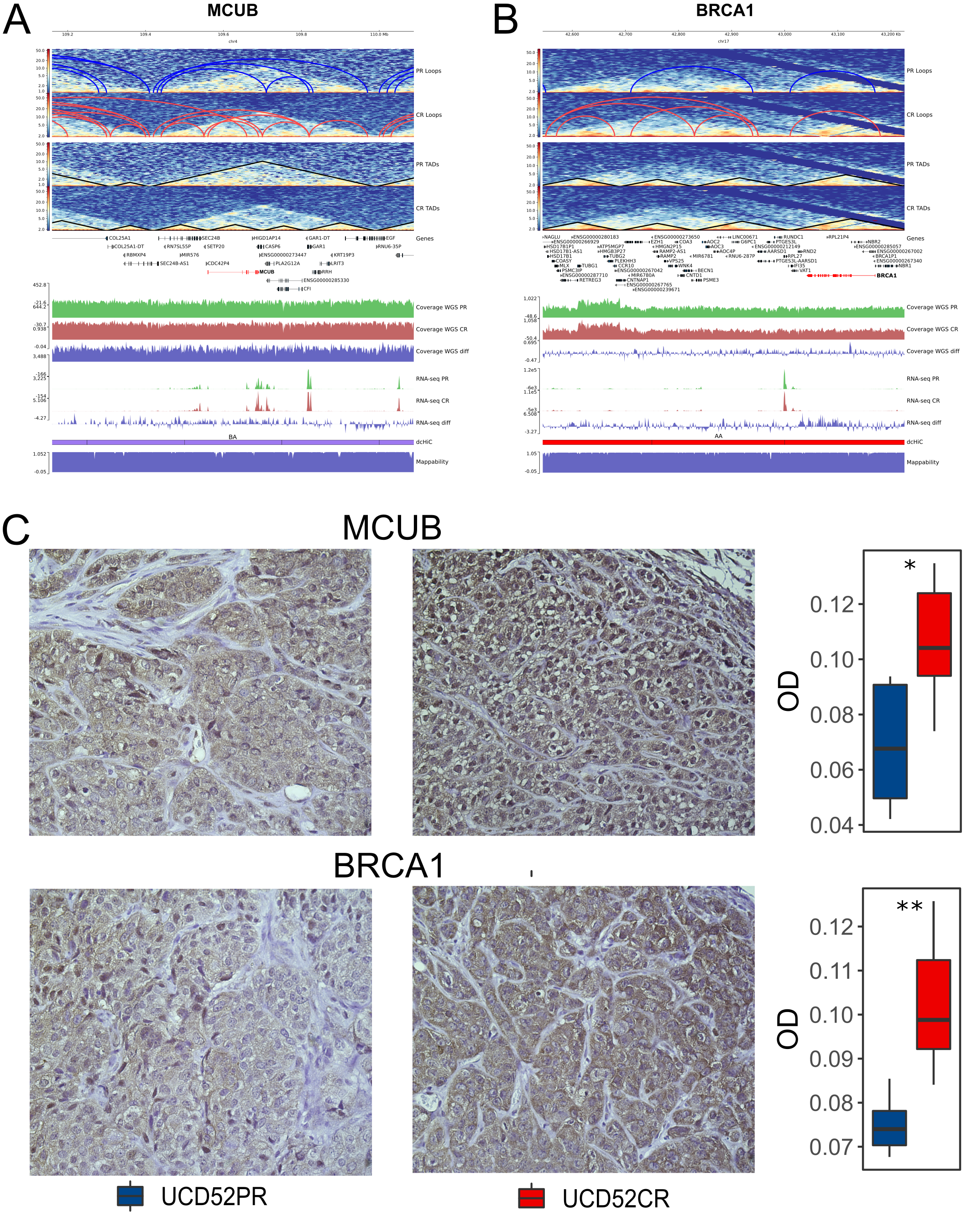
 **Figure S7. Examples of genes activated in carboplatin resistance and supported by multiple evidences.** (A) MCUB, mitochondrial calcium uniporter dominant negative subunit beta; (B) BRCA1, BRCA1 DNA repair associated; (C) Quantification of MCUB and BRCA1 expression in UCD52 primary tumor (UCD52PR) and carboplatin-resistant (UCD52CR) conditions (n = 6, two replicates x three image areas). */** - t-test p-value <0.05/0.01, respectively.


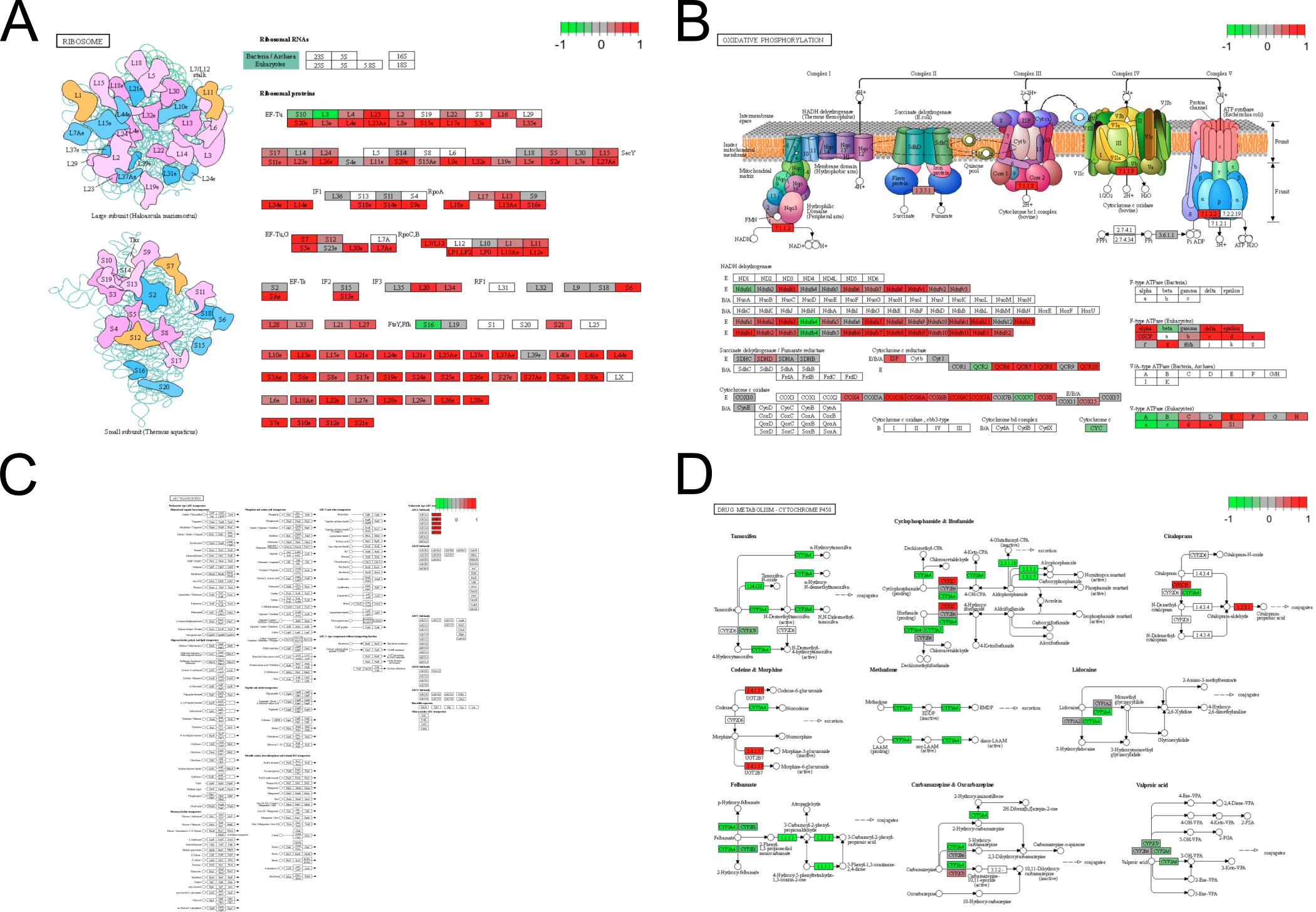
 **Figure S8. Selected KEGG pathways.** (A) Ribosome and (B) Oxidative phosphorylation pathways, red/green indicate gene expression level up-/downregulated in CR, respectively (RNA-seq-colored). (C) ABC transporter pathway, red indicates genes amplified in CR (WGS-colored). (D) Drug metabolism pathway, red/green indicate genes overlapping chromatin switching into active/inactive state, respectively (dcHiC-colored).
